# Seamless Integration of Laser-Induced Papertronics with Parafilm-Based Microfluidics as a Versatile Paper-Based Electroanalytical Platform

**DOI:** 10.1021/acsami.5c09316

**Published:** 2025-06-26

**Authors:** Lingyin Meng, Danfeng Cao, Jonas Oshaug Pedersen, Grzegorz Greczynski, Vladyslav Rogoz, Warakorn Limbut, Mats Eriksson

**Affiliations:** † Division of Sensor and Actuator Systems, Department of Physics, Chemistry and Biology, 4566 Linköping University, Linköping 581 83, Sweden; ‡ Laboratory of Organic Electronics, Department of Science and Technology, 196410 Linköping University, Norrköping 601 74, Sweden; § Division of Biophysics and Bioengineering, IFM, Linköping University, Linköping 581 83, Sweden; ∥ Thin Film Physics Division, Department of Physics, Chemistry and Biology (IFM), 4566 Linköping University, Linköping SE-581 83, Sweden; ⊥ Center of Excellence for Trace Analysis and Biosensor, Prince of Songkla University, Hat Yai, Songkhla 90110, Thailand

**Keywords:** cellulose paper, laser, paper electronics, microfluidics, sensing and biosensing

## Abstract

The widespread use of nonrenewable materials in point-of-care (PoC) electroanalysis, such as test strips with electronic meters, has inadvertently contributed to electronic waste. Paper, traditionally used as a passive substrate, offers a renewable alternative as a sustainable and versatile electroanalytical platform for on-site analysis. Here, we present the fabrication and integration of laser-induced electronic components and Parafilm-based microfluidics on a single sheet of paper as a versatile electroanalytical platform for both aqueous and organic systems. Using a flame retardant and laser treatment, we enable a direct conversion of passive cellulose paper into laser-induced graphite (PLIG), allowing for the fabrication of conductive pathways and various electronic components with customized geometries on a single sheet of paper, a process termed laser-induced papertronics. Microfluidic channels were then successfully patterned by hot-pressing hydrophobic Parafilm into hydrophilic cellulose paper (paper-para) at a low temperature (60 °C) for just 15 s, achieving a submillimeter resolution of ∼0.45 mm. The resulting paper-para demonstrated compatibility with a wide range of aqueous solutions and organic solvents. This process facilitates the seamless integration of laser-induced papertronics with Parafilm-based microfluidics on a single monolithic paper sheet, denoted microfluidic PLIG (μPLIG), preserving both the structural integrity and electrochemical performance of the papertronics as well as the fluidic character of the Parafilm-based paper microfluidics. Demonstrative applications include pH sensing with a sensitivity of −40.3 mV pH^–1^, lactate biosensing with a sensitivity of 0.92 μA mM^–1^, and Vitamin D3 detection in ethanol mixtures exhibiting a linear range of 5–65 μM, indicating the platform’s compatibility and versatility for sensor applications in both aqueous and organic systems. This study establishes a foundation for a uniquely integrated, cost-effective, and environmentally friendly electroanalytical platform, μPLIG, uniting paper-based LIG electronics and Parafilm-based microfluidics on a single disposable substrate.

## Graphical abstract

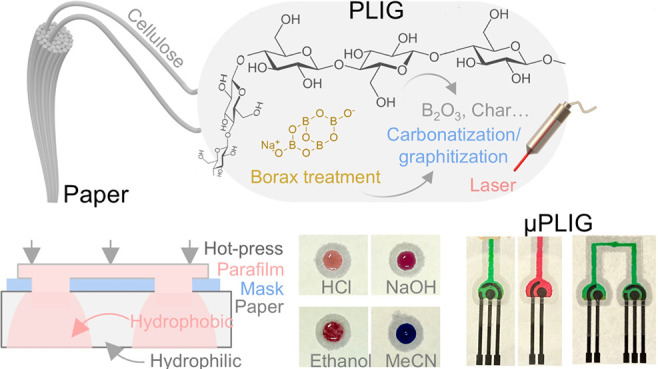

## 1 Introduction

Electroanalytical point-of-care (PoC) testing, which utilizes disposable test strips in conjunction with electronic meters, has been revolutionary for on-site disease diagnostics and health management, particularly in applications such as blood glucose monitoring.^1^ However, these test strips are typically fabricated from nonrenewable materials, including metals and carbon allotropes on ceramics or plastics.^2^ Lignocellulosic biomass, abundant and renewable, offers cellulose paper as an eco-friendly and affordable material due to its unparalleled affordability, biodegradability, and biocompatibility.^3^ In recent years, there has been a surge of interest in leveraging disposable cellulose paper^3^ as a substrate for building electronic devices, known as papertronics,^4,5^ for applications including sensors, displays, and energy storage.^4,5^ So far, paper primarily serves as a passive substrate for externally additive printing of conductive materials (e.g., metals and metal oxides, carbon allotropes, and conducting polymers).^6–8^ Therefore, to unlock the full potential of papertronics, an important step would be to directly convert the paper itself into conductive paths and integrate it with sample manipulation to realize analytical properties on a single monolithic and disposable paper sheet.

Recently, laser processing has been employed for efficient and maskless construction of conductive laser-induced graphene/graphite (LIG) from aromatic ring-rich polymeric materials, such as polyimide (PI), as a versatile electrode platform for disposable, flexible, or wearable sensors.^9–14^ Beyond this, LIG derived from PI has been further integrated with microfluidics to fabricate sensing devices.^15–19^ However, such systems typically rely on synthetic polymer substrates and involve complex multilayered assembly steps. For instance, the integration often includes photolithography of polydimethylsiloxane (PDMS),^16^ hot embossing of poly­(methyl methacrylate) (PMMA),^17^ adhesive bonding of glass/acrylic,^18^ or chemical modification of LIG wettability combined with external 3D-printed housings,^19^ resulting in a multilayered structure that often requires complex assembly and lacks eco-disposability. Despite their versatility, these LIG-based systems are also limited by their use of synthetic, nonbiodegradable PI. In contrast, cellulose paper offers a sustainable and hygroscopic alternative substrate that can simultaneously serve as the basis for both the paper-based LIG (PLIG) sensing components and the microfluidic compartments. However, unlike PI, laser processing on cellulose paper typically leads to ablation and combustion erosion. To avoid polymeric combustion, flame retardant treatment has recently been used to endow polymeric materials with flame retardation.^20^ Therefore, the combination of flame retardant treatment (e.g., borax, boric acid, commercial phosphate-based flame retardant, etc.) and laser processing for cellulose paper has enabled the direct fabrication of PLIG in various patterns (e.g., capacitors and sensing and biosensing electrode systems) on paper platforms.^21–25^

On the other hand, microfluidic paper-based analytical devices (μPADs) have garnered significant interest over the past decade as an analytical platform due to their passive capillary transportation of liquids and flexibility.^26^ These devices involve patterning of paper with hydrophobic barriers to define hydrophilic channels and zones,^26^ thus allowing the manipulation of tiny volumes (∼μL) of liquid without the need for external pumps. Electrochemical readouts based on conductive electrodes have been further integrated with μPADs, forming electrochemical paper-based analytical devices (ePADs) capable of quantitative readouts with enhanced sensitivity and broad analytical applications.^26,27^ The fabrication of ePADs is usually achieved by microfluidic patterning of hydrophobic barriers and subsequent screen/stencil/inkjet printing of conductive inks on a paper substrate. Recently, Bezinge et al.^28^ introduced a new paper-based electrofluidic system for diagnostic bioassays, integrating graphenic electrodes produced by laser-induced pyrolysis of cellulose (i.e., PLIG) with fluidic channels patterned through wax lamination. While wax-based μPADs are compatible with aqueous solutions, they usually exhibit incompatibility with organic solvents due to the solubility and structural instability of the wax in nonaqueous environments. This can lead to wicking through or degradation of the hydrophobic barriers.^29,30^ Consequently, this limitation restricts the versatility of ePADs, particularly for sensing applications involving organic solvents or mixed-phase samples where target analytes are not soluble in an aqueous system.^31,32^ Moreover, the wax patterning technique requires relatively high-temperature heating (e.g., 110 °C)^28^ and prolonged baking for effective wax penetration, similar to previously reported protocols such as wax printing baked at 120 °C for 120 s,^29^ thermal transfer printing baked at 90 °C for 15 min,^30^ and laser printing baked at 200 °C for 60 min.^33^

Parafilm, a blend of waxes and polyolefins, has been recognized as an alternative for paper microfluidic patterning via embossing/hot-pressing.^34,35^ It is worth noting that the compatibility of PLIG with hot-pressing techniques has not been evaluated. Given the porous nature of PLIG, there is a concern that it may not be robust enough to withstand microfluidic patterning methods, potentially leading to the disruption of structural integrity and a subsequent decline in electrochemical performance. To date, the integration of laser-induced papertronics with Parafilm-based paper microfluidics compatible with organic solvents on a single sheet of paper has not yet been achieved.

Herein, we present a seamless integration of laser-induced papertronics with Parafilm-based microfluidics on a single monolithic and disposable paper sheet, creating a sustainable and versatile electroanalytical platform compatible with sensing in both aqueous and organic systems. Our approach combines flame retardants and laser treatment to directly convert cellulose paper into laser-induced graphite (PLIG) with customizable papertronic components. Microfluidic channels, with submillimeter resolution, are patterned on the paper substrate by hot-pressing hydrophobic Parafilm at a low temperature of 60 °C for just 15 s, which circumvents the prolonged baking at relatively high temperatures typically required in previous methods. Such seamless integration of microfluidics with PLIG (μPLIG) preserves both the structural integrity and electrochemical performance of the papertronics while maintaining the fluidic properties of the Parafilm-based microfluidics. In contrast to previous LIG-based microfluidic systems that often rely on synthetic polymer substrates (e.g., polyimide) and multimaterial assembly involving PDMS, PMMA, or 3D-printed components, our μPLIG platform achieves monolithic integration without additional structural layers or adhesives. Notably, our platform demonstrates compatibility with both aqueous and organic solvents, expanding the scope of electroanalytical applications from sensing and biosensing in aqueous solutions to organic compound detection in organic solvent systems. This study paves the way for developing a cost-effective, disposable, and versatile electroanalytical platform for on-site analysis.

## 2 Experimental Section

### 2.1 Materials

Whatman qualitative filter paper (Grade 1), sodium tetraborate (borax), Parafilm M sealing film, potassium ferricyanide (K_3_[Fe­(CN)_6_]), potassium ferrocyanide (K_4_[Fe­(CN)_6_]), iron chloride (FeCl_3_), potassium chloride (KCl), polyaniline (emeraldine base) (PANi), Nafion, lithium perchlorate (LiClO_4_), and sodium lactate were purchased from Sigma-Aldrich. Lactate oxidase (LOx, 106 U/mg) was purchased from Toyobo (Japan). Polyester films (3M, PP2500) were purchased from 3M (USA). Silver/silver chloride (Ag/AgCl) was purchased from DuPont (USA). All solutions were prepared with deionized water from a Milli-Q system. Phosphate-buffered saline (PBS, 0.1 M, pH 6.4) was prepared by mixing dipotassium hydrogen phosphate and potassium dihydrogen phosphate.

### 2.2 Borax and Laser Treatment for PLIG Fabrication

To impart flame retardancy to the paper substrate, the filter paper was immersed in a 0.1 M borax solution for 10 min and dried overnight under ambient conditions, as previously reported,^24^ which was denoted as paper-borax. PLIG was fabricated by irradiating the paper-borax using a computer-controlled HL40–5g CO_2_ laser (10.6 μm, Full Spectrum Laser LLC, USA) under a defocus length of 5 mm (distance to the work plane of the substrate below the laser focal plane), based on a previous report.^22^ The resulting PLIG fabricated under defocus conditions was denoted as PLIG-De. Laser parameters, including power (*P*, full power of 40 W) and scan speed (*S*, full scan speed of 80 in. s^–1^), were optimized by adjusting them in the range of 20–42.5% (denoted as P20–42.5) and 10–100% (denoted as S10–100), respectively. Thus, S10P25 denotes laser treatment at 10% of the maximum scan speed and 25% of the maximum power. Various papertronic patterns were fabricated based on the optimized conditions of PLIG-De, including a strip resistor, an interdigital capacitor, an antenna, as well as 2- and 3-electrode systems. Beyond the defocus conditions, PLIG was also fabricated under focus conditions (PLIG-Fo) using the same laser parameters as PLIG-De, except for adjusting the laser height into the focal plane. Moreover, laser treatment was applied to paper-borax under two consecutive scans (i.e., one defocus scan followed by one focus scan), with the resulting PLIG denoted as PLIG-DeFo.

### 2.3 Hot-Press Patterning of Microfluidics on Paper

The microfluidics, featuring patterned hydrophobic barriers to define hydrophilic channels and zones, were fabricated on paper by hot-pressing hydrophobic Parafilm into hydrophilic cellulose paper through a mask. This process was conducted by using a hot-pressing machine (3.8 MPa, KP-4, LTQ Vapor). The mask, designed with various patterns (such as for a single Parafilm hydrophobic channel, single hydrophilic channel, three-channel, detection zone), was cut from a polyester film (thickness of ∼100 μm) using a cutting plotter (Brother ScanNCut CM900). Various layers were aligned in the sequence of Parafilm, mask, and paper from top to bottom. The pressing parameters were optimized by adjusting the heating temperature in the range of 50–70 °C and the pressing time of 5–30 s, respectively, to ensure the melting and infusion of Parafilm from the front to the back of the paper.

### 2.4 Integration of Microfluidics with Papertronic Components for μPLIG

Laser-induced papertronics with specific patterns (2- and 3-electrode systems) were fabricated via borax and laser treatment, followed by hot-pressing of the Parafilm to define the microchannels and detection zones. The compatibility of PLIG with hot-pressing pressure was first evaluated by CV and EIS using a typical 3-electrode system as a model in 5 mM Fe­(CN)_6_^3–/4–^ in 0.1 M KCl against a glass Ag/AgCl (3 M KCl) reference electrode. Then, the impact of the hot-pressed hydrophobic Parafilm on the PLIG was evaluated by hot-pressing two flow channels (3 mm) perpendicular to a PLIG strip (5 mm), with one channel blocked in the middle of the PLIG strip by Parafilm. μPLIG, integrating PLIG electrode systems and Parafilm-based microfluidics, was prepared by hot-press patterning microchannels and detection zones over 2-, 3-, and dual 3-/2- electrode systems. Reproducibility and storage stability were investigated based on anodic peak current (Ipa) and peak-to-peak separation (Δ*E*) values obtained by cyclic voltammetry (CV) at the 3-electrode μPLIG in 5 mM Fe­(CN)_6_^3–/4–^ in 0.1 M KCl against a glass Ag/AgCl (3 M KCl) reference electrode. Bending and twisting effects on the electrochemical performance of the μPLIG were assessed by multiple-step chronoamperometry (CA) using a 3-electrode μPLIG in 5 mM Fe­(CN)_6_^3–/4–^ in 0.1 M KCl against an internal Ag/AgCl reference electrode (stencil-coated Ag/AgCl ink applied using a paintbrush). CA current–time curves were recorded under open-circuit potential (0.165 V), as well as oxidation peak potential (0.250 V) and reduction peak potential (0.080 V) collected from CV.

### 2.5 Demonstrative Sensing and Biosensing Applications

To demonstrate the versatility of the μPLIG as an electroanalytical platform, three representative proof-of-concept applications were investigated: 1) potentiometric pH sensing, ionic sensing of H^+^ concentration that is important for quality control, environmental, and biomedical analysis;^36^ 2) amperometric lactate biosensing, an important biomarker relevant in biomedical diagnostics (e.g., sepsis monitoring, anaerobic metabolism, and fitness testing);^37^ and 3) voltammetric Vitamin D3 detection, which is important for adequate nutritional supplementation,^38^ showcasing the platform’s compatibility with nonaqueous or mixed-phase systems. The reference electrode area of all the μPLIGs was stencil-coated with Ag/AgCl ink using a paintbrush, followed by drying under ambient conditions for 30 min. For pH sensing, the working electrode area of a 2-electrode μPLIG was functionalized by drop-casting 10 μL of PANi (5 mg mL^–1^). The pH sensor response was recorded as the potential difference between the working and reference electrodes in Britton–Robinson buffer with 0.1 M KCl, with varied pH of 6–9. For lactate biosensing, the working electrode area of a 3-electrode μPLIG was functionalized by drop-casting 2 μL of Prussian blue-LOx-Nafion composites onto the working electrode. Specifically, Prussian blue was synthesized by mixing equal volumes of 5 mM K_4_[Fe­(CN)_6_] and 5 mM FeCl_3_ in 10 mM HCl, followed by sonication for 30 min, washing with ethanol, and drying at 70 °C for 2 h. The Prussian blue-LOx-Nafion composite was then prepared by mixing Prussian blue (10 mg/mL), LOx (40 mg mL^–1^), BSA (10 mg mL^–1^), and Nafion (1%) in water. Amperometric lactate biosensing was performed under static conditions in 0.1 M PBS with various concentrations of lactate under an operational potential of −0.2 V. For Vitamin D3 detection, a 3-electrode μPLIG was used without further functionalization. The voltammetric response was recorded by differential pulse voltammetry (DPV) in 0.1 M LiClO_4_ in a 50% ethanol/50% water mixture (v/v). DPV analysis was performed over the potential range of 0.4–1 V, a scan rate of 5 mV s^–1^, a pulse time of 50 ms, and a pulse amplitude of 50 mV.

### 2.6 Characterization and Measurements

Scanning electron microscopy (SEM) images of paper, paper-borax, and the as-prepared PLIG (without water rinsing after laser treatment) were recorded using a Zeiss-Sigma 500 Gemini electron microscope (Zeiss, Germany). The chemical composition was determined by energy-dispersive X-ray spectroscopy (EDX, Oxford Instruments) after SEM image acquisition. Thermogravimetric analysis (TGA) and derivative thermogravimetric (DTG) measurements of paper, paper-borax, and the as-prepared PLIG were carried out in the range of 25–590 °C using a TGA Q500 (TA Instruments). PLIG samples were rinsed with deionized water to remove any residues prior to all characterization and measurements listed below. Sheet resistance was measured with a Jandel RM3000 station (Jandel Engineering Limited, UK). Fourier transform infrared (FTIR) spectra were obtained via a VERTEX spectrometer (Bruker, USA) equipped with an attenuated total reflection (ATR) measuring cell. Raman spectroscopy was conducted using an Andor Kymera 328i Raman spectrometer connected to a Nikon air objective (60×), using an excitation wavelength of 532 nm and an output power of 10 mW. The spectrometer was equipped with a diffraction grating of 600 lines/mm and a thermoelectrically cooled (−80 °C) EMCCD camera (Andor Newton DU970P-BVF). X-ray photoelectron spectroscopy (XPS) analyses were conducted with an Axis Ultra DLD instrument (Kratos Analytical, UK) equipped with a monochromatic Al Kα X-ray radiation source (*h*ν = 1486.6 eV). The base pressure was better than 1.1 × 10^–9^ Torr (1.5 × 10^–7^ Pa), while the anode was operated at 150 W (10 mA, 15 kV). The sample area analyzed was 0.3 × 0.7 mm^2^. The instrument binding energy scale was calibrated using sputter-etched Au, Ag, and Cu samples as previously reported.^39^ A charge neutralizer was used when recording all spectra. Charge referencing was done by setting the C–C peak of the C 1s spectra from paper and paper-borax (as insulating samples) to 285.0 eV, assuming that the contribution of adventitious carbon to the C 1s spectra is negligibly small, such that related problems can be avoided.^40,41^ Quantification and deconvolution were performed with Casa XPS software (Casa Software Ltd.). Wettability was evaluated by measuring the static contact angle through a KSV CAM200 semiautomatic drop-shape analysis system (KSV Instrument, Helsinki, Finland). Optical microscopic images were obtained using an Lx-0624 Zoom Stereo Binocular Compound Microscope equipped with a Dino-eye camera. A CompactStat potentiostat (Ivium, Netherlands) was used for electrochemical performance characterization and analysis measurements, including CV, electrochemical impedance spectroscopy (EIS), CA, potentiometry, and DPV.

## 3 Results and Discussion

### 3.1 Direct Conversion of Paper into Papertronic Components

The direct conversion of lignocellulosic paper is enabled via the synergistic effect of flame-retardant borax treatment and laser processing, as schematically illustrated in Figure 1a. Borax enhances flame retardancy,^42,43^ while laser irradiation induces localized heating via the photothermal effect, leading to controlled carbonization and graphitization of cellulose.^24,25^ The optimum combination of laser scan speed (*S*) and laser power (*P*) was S10P25, based on a compromise between conductivity and PLIG mechanical properties (adhesion, no burn-through); see Figures S1 and **S2**. This setting was chosen for the following PLIG fabrication. Figure 1b displays the fabrication of papertronic components with customized patterns via laser processing of paper-borax in a defocus mode, including a resistor, an interdigital capacitor (IDC), an antenna, and a 3-electrode system. In contrast, laser processing of the original paper substrate without flame-retardant treatment results in the ablation of cellulose due to pyrolytic decomposition (Figure 1c).

**Figure 1:**
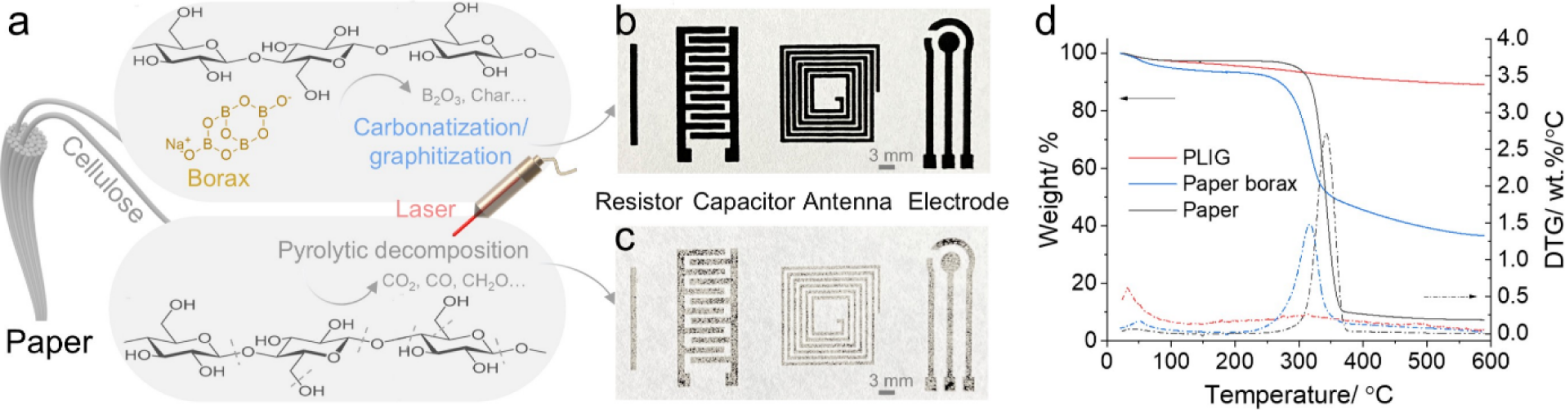
Direct conversion of paper into papertronic components on a single sheet of paper via flame retardant and laser treatment. (a) Schematic illustration of the borax and laser treatment of cellulose paper. Digital photographs of various designs of the conductive area by laser processing of paper-borax (b) and original paper (c) under S10P25 laser treatment in defocus mode. (d) TGA and DTG measurements of paper, paper-borax, and PLIG-De.

The effect of the borax and laser treatment was evaluated by measuring the thermal stability of paper, paper-borax, and PLIG with TGA, with the corresponding TGA/DTG results shown in Figure 1d. The weight of paper began to decrease at ∼270 °C owing to thermal degradation, with the maximum degradation rate reaching 2.71 wt %/°C at 342 °C, and a final char residue of 7.3% at 590 °C. This is consistent with the apparent ablation of the patterns from the paper surface in Figure 1c. After the borax treatment, the initial thermal degradation temperature and maximum degradation rate temperature for paper-borax decreased to ∼240 and 317 °C, respectively. This shift is attributed to the stimulated thermal degradation and char formation effect caused by the flame retardant.^44^ Nevertheless, the maximum degradation rate decreased to 1.47 wt %/°C, and the char residue increased to 36.6%, indicating that borax enhances the paper’s flame retardancy and its tendency to form char under thermal treatment. Only a slight weight loss can be observed for PLIG due to moisture and volatile evaporation, with 89.9% weight retained. These results demonstrate an efficient, cost-effective, and mask-free generation of papertronic components with customized geometries directly on a single sheet of paper, denoted as laser-induced papertronics.

Previous studies have indicated that defocus/focus combinations and multiple laser scans impact the yield and physicochemical properties of graphitic materials derived from various polymeric precursors.^22^ To optimize the physicochemical properties of PLIG from borax and laser treatment, we further investigated defocus (PLIG-De), focus (PLIG-Fo), and consecutive defocus and focus conditions (PLIG-DeFo). The physicochemical properties of the various PLIG samples were examined using SEM, EDX, FTIR, TGA, Raman spectroscopy, and XPS. Figure 2a displays top-view SEM images of paper (i), paper-borax (ii), PLIG-De (iii), PLIG-Fo (iv), and PLIG-DeFo (v) at two different magnifications (the corresponding fiber diameter distribution is summarized in Figure S3). The original paper (i) consists of cellulose microfibers (diameter distribution of 4.2–30.8 μm) that overlap with each other into a network and present a smooth surface. The flame-retardant treatment of the paper (paper-borax (ii)) did not alter the morphology noticeably. The PLIG produced under mild laser conditions (e.g., S10P22.5 in defocus mode, Figure S3) retains the fibrous structure of cellulose (diameter distribution of 6.6–39.4 μm) but introduces numerous microholes with the generation of nanofibers (diameter distribution of 57.5–363 nm) due to photothermal decomposition. The increase of laser power (S10P25) further decomposes the cellulose microfibers (diameter distribution of 3.8–30.3 μm) and nanofibers (diameter distribution of 56.4–306 nm), converting them into a graphitic material with graphitic snippet residues on the fiber edges, as shown for PLIG-De (iii). When the laser is focused (PLIG-Fo (iv)), the cellulose fibrous structure is more severely damaged, leading to the formation of granular graphitic particles on the surface. This is ascribed to the higher laser radiation energy density in focus mode compared to defocus mode and thus increased pyrolytic decomposition and carbonization/graphitization. The PLIG-DeFo (v), resulting from two consecutive defocus–focus conditions, shows a well-preserved fibrous structure (diameter distribution of 2.9–24.6 μm) and a graphitic structure, indicating a cumulative effect of both defocus and focus by retaining the fibrous structure while enhancing the laser conversion efficiency. Successful conversion is further supported by the disappearance of abundant oxygen-containing functional groups, as observed in the FTIR spectra (Figure S4), and a pronounced increase in carbon content, as shown in EDX spectra (Figure S5). It should be noted that the rise in boron and sodium in PLIG compared to paper-borax might be ascribed to an accumulation of borates on the surface after the laser treatment, which is consistent with our previous report of an accumulation of Na-containing inorganic compounds in lignin-derived LIG.^13^

**Figure 2:**
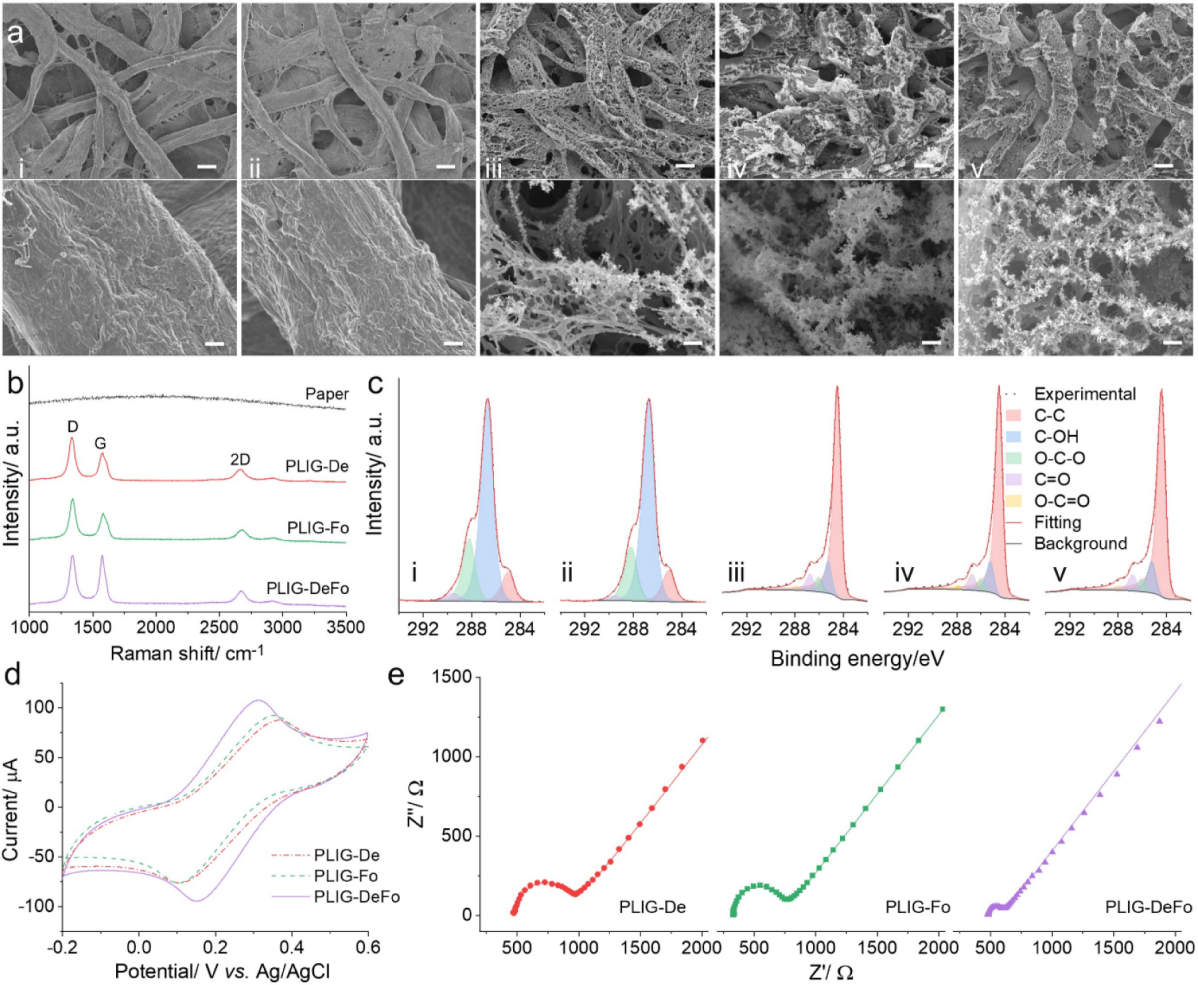
Optimization of PLIG by defocus/focus and multiple laser scans. (a) SEM images of paper (i), paper-borax (ii), PLIG-De (iii), PLIG-Fo (iv), and PLIG-DeFo (v) in low magnification (top) with a scale bar of 20 μm as well as in high magnification (bottom) with a scale bar of 2 μm (paper and paper-borax) and 1 μm (PLIGs). (b) Raman spectra. (c) XPS C 1s spectra of paper (i), paper-borax (ii), PLIG-De (iii), PLIG-Fo (iv), and PLIG-DeFo (v). CV (d) and EIS (e) plots for PLIG-De, PLIG-Fo, and PLIG-DeFo in Fe­(CN)_6_^3‑–/4‑–^ in 0.1 M KCl.

In the Raman spectra (Figure 2b), no detectable bands are observed for the paper. After laser treatment, all PLIG spectra present three main bands, including a disorder-induced D band at 1340 cm^–1^, a graphitic characteristic G band for sp^2^ bonded carbon at 1576 cm^–1^, and a 2D band as an overtone of the D-band at 2670 cm^–1^.^45^ The peaks are also quite broad, especially noticeable by the fact that the D’ band (at ∼1608 cm^–1^) appears as a shoulder of the G band and not as a separate band. These features appear because of disorders in the graphitic structure. The D to G intensity ratio (*I*_D_/*I*_G_) is indicative of the level of disorder in the graphitic material.^45^ Among all of the PLIG samples, PLIG-DeFo possesses the lowest *I*_D_/*I*_G_ value of 1.00 compared to PLIG-De (1.44) and PLIG-Fo (1.33), indicating a lower level of disorder and a higher degree of graphitization in PLIG-DeFo.

The effects of the flame-retardant treatment and laser processing were further investigated by XPS. The XPS survey spectra (Figure S6) depict C 1s (285 eV) and O 1s (532 eV) as prevalent in all paper and PLIG samples. The appearance of Na 2s and B 1s peaks in the region between 40 and 220 eV (Figure S7a,b) corroborates the successful modification of the paper with borax. Subsequent laser processing contributed to a significant increase of the C content, rising from ∼60% for paper and paper-borax to over 90% for the various PLIG samples (Table S1), which is consistent with the EDX results. Figure 2c shows the high-resolution C 1s spectra deconvoluted into different carbon-containing functional groups, with detailed percentages listed in Table S1. Paper (i) and paper-borax (ii) exhibit similar features with primary C–C and C–O (C–OH and O–C–O) functional groups. A minor deconvoluted peak observed in the high binding energy region, attributed to carbocyclic C (not expected in these samples), is likely due to inhomogeneous charging artifacts commonly encountered in cellulose XPS spectra.^41^ Notably, the XPS spectra of paper and paper-borax were obtained by measuring the edge spot (close to the conductive contact of the sample holder) of the samples, which reduced charging artifacts (the difference between the middle and edge spot is discussed in connection with Figure S8).^39^ In contrast, laser processing of paper-borax results in the cleavage of oxygen-containing groups, with a remarkably increased C–C percentage of over 73% for the various PLIG samples compared with those of paper (9.86%) and paper-borax (10.73%).

The electrochemical performance of PLIG-De, PLIG-Fo, and PLIG-DeFo was investigated by CV and EIS. Figure 2d displays a pair of quasi-reversible redox peaks, with anodic peak current (Ipa) and peak-to-peak separation (Δ*E*) values summarized in Table S2. The PLIG-De exhibited an Ipa value of 87.3 μA and a Δ*E* of 257 mV. For PLIG-Fo, the Ipa increased to 92.3 μA, approximately 1.06 times higher than that of PLIG-De, while the Δ*E* decreased slightly to 245 mV, indicating an improved charge transfer rate. PLIG-DeFo showed an Ipa of 107.4 μA, which is 1.23 and 1.16 times higher than those of PLIG-De and PLIG-Fo, respectively. Additionally, PLIG-DeFo demonstrated the lowest Δ*E* value of 149 mV among all the PLIG electrodes. Furthermore, the EIS Nyquist plots (Figure 2e) reveal a decrease in the semicircle diameter (representing the charge transfer resistance, *R*_ct_) from PLIG-De (468.0 Ω) to PLIG-Fo (413.7 Ω) and to PLIG-DeFo (122.4 Ω). The heterogeneous electron transfer rate constant (*k* ^0^) of each PLIG electrode was calculated from the measured *R*_ct_ using the following equation:^46^

$$R_{ct}=RT/n^{2}F^{2}Ak^{0}C$$

where *R*, *T*, *n*, *F*, *A*, and *C* are the gas constant (8.314 J mol^–1^ K^–1^), absolute temperature (298 K), number of electrons transferred (*n* = 1 here), the Faraday constant (96 485 C/mol), electrode surface area (0.071 cm^2^), and the concentration of the redox probe (5 mM), respectively. The calculated *k* ^0^ values were 1.60 × 10^–3^, 1.81 × 10^–3^, and 6.12 × 10^–3^ cm s^–1^ for PLIG-De, PLIG-Fo, and PLIG-DeFo, respectively. These values correlate well with the increasing trend in electrical conductivity observed for the corresponding PLIG electrodes: 1.93 S cm^–1^ for PLIG-De, 2.17 S cm^–1^ for PLIG-Fo, and 2.94 S cm^–1^ for PLIG-DeFo (Figure S9). These electrochemical performance results indicate that PLIG resulting from the DeFo mode is superior to PLIG produced by a single defocus or focus in terms of electrochemical kinetics. Consequently, the DeFo mode was chosen as the optimal method for the fabrication of laser-induced papertronics hereinafter.

### 3.2 Hot-Press Patterning of Microfluidic Channels on the Paper Substrate

Leveraging the intrinsic capillary wicking action of fibrous cellulose paper, microfluidic channels were patterned by hot-pressing hydrophobic Parafilm into the hydrophilic cellulose paper for fluidic manipulation. The Parafilm was melted by plate heating, allowing it to penetrate throughout the paper via pressing, forming hydrophobic barriers while leaving the surrounding area hydrophilic. The hot-pressing parameters were optimized with a relatively low temperature of 60 °C and a short baking time of 15 s, resulting in the successful melting and infusion of Parafilm from the front to the back of the paper (Figure S10). Various types of patterning were explored: (1) patterning a hydrophobic barrier via directly pressing a Parafilm strip into the paper (Figure 3a), (2) patterning a hydrophobic barrier via pressing Parafilm into the paper through a mask (Figure 3b), and (3) patterning a hydrophilic channel via forming two adjacent Parafilm barriers through a mask (Figure 3c).

**Figure 3:**
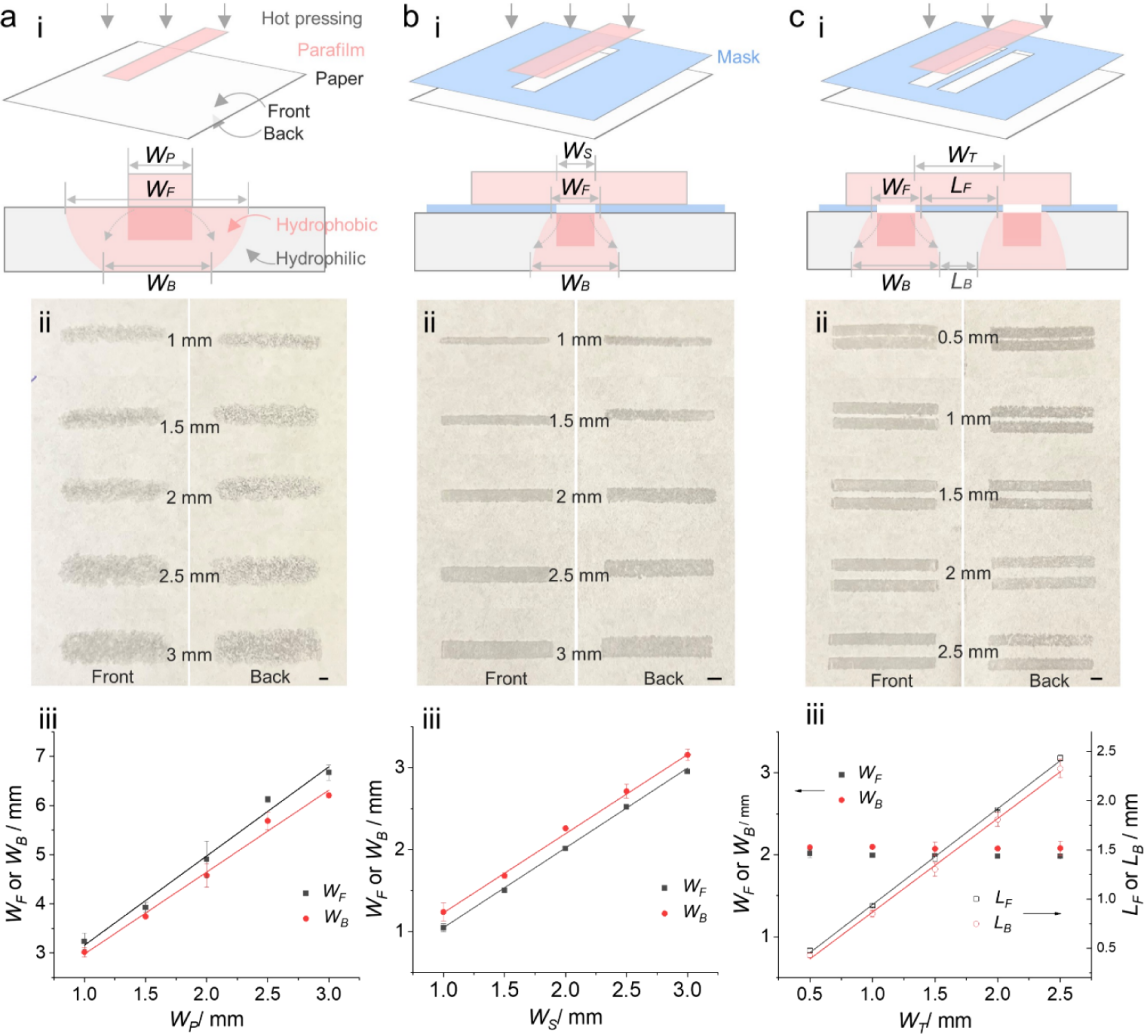
Hot-press patterning of microfluidic channels on the paper substrate. (a) Patterning a hydrophobic barrier via directly pressing a Parafilm strip, (b) patterning a hydrophobic barrier via pressing Parafilm through a mask, and (c) patterning a hydrophilic channel via forming two adjacent Parafilm barriers through a mask, with (i) diagrammatic illustration of pressing and infusion/spreading processes, (ii) digital photograph of the resulting paper-para in both front and back sides (scale bar of 2 mm), and (iii) plots of the width of the hydrophobic barrier (*W*) or hydrophilic channel (*L*) as a function of the Parafilm strip width (*W*_P_), pattern width in the mask (*W*_S_) or template width (*W*_T_) between the two adjacent strips on the mask.

Figure 3ai illustrates the direct pressing of a designed Parafilm strip into paper, showing both a horizontal spreading effect and a vertical infusion effect of the molten Parafilm from the paper-front to the paper-back. Consequently, the width of the hydrophobic barrier on the paper-front (*W*_F_) and paper-back (*W*_B_) is correlated with the width of the Parafilm strip (*W*_P_). As shown in the digital photograph of the paper-para-front/back in Figure 3aii, Parafilm infused from the paper-front to the paper-back, with an increasing trend of *W*_F_ and *W*_B_ as *W*_P_ expands from 1 to 3 mm. However, this process results in uneven dispersion of Parafilm with a diffuse edge (detailed microscopic images are shown in Figure S11), which is ascribed to the fast horizontal spreading of Parafilm via a capillary flow in the fibrous cellulose paper. In addition, the resulting *W*_F_ and *W*_B_ are proportional to *W*_P_, as shown in Figure 3aiii. The equations below indicate that *W*_F_ is greater than *W*_B_, likely due to the longer horizontal spreading time of the molten Parafilm on the paper-front side compared with the paper-back side. For instance, with a 1 mm Parafilm strip, *W*_F_ was measured to be 3.16 mm, which is 1.06 times larger than *W*_B_ (2.98 mm).

$$W_{F}=1.81\times W_{P}+1.35(R^{2}\;\mathrm{of}\;0.988)$$

$$W_{B}=1.67\times W_{P}+1.32(R^{2}\;\mathrm{of}\;0.991)$$

The use of a mask with a strip pattern, sandwiched between a Parafilm strip and the paper substrate, can significantly reduce the horizontal spreading of molten Parafilm, as illustrated in Figure 3bi. This method produces a sharp, well-defined edge on the paper-front, while the paper-back displays a slightly diffuse edge, as seen in Figure 3bii and the microscopic images in Figure S11. Interestingly, Figure 3biii reveals that the spreading trend of molten Parafilm on the paper-front and paper-back is the opposite of what was observed in direct patterning. The values of *W*_F_ and *W*_B_ are described by the following equations below. With a 1 mm width of the strip pattern in the mask (*W*_S_), the resulting *W*_F_ value is 1.05 mm, which is smaller than the *W*_B_ value of 1.23 mm. This difference can be attributed to the mask restricting horizontal spreading of the molten Parafilm on the paper-front side. Additionally, the resulting *W*_F_ and *W*_B_ values are 0.33 and 0.41 times smaller than those obtained through direct pressing of Parafilm strips, respectively. These results indicate improved resolution in hydrophobic barrier patterning on paper when using a mask during the hot-pressing process.

$$W_{F}=0.97\times W_{S}+0.08(R^{2}\;\mathrm{of}\;0.998)$$

$$W_{B}=0.97\times W_{S}+0.26(R^{2}\;\mathrm{of}\;0.999)$$

Beyond the fabrication of the hydrophobic barrier described in Figure 3a,b, hydrophilic channels can also be formed between two adjacent hydrophobic barriers via hot-pressing with a mask, as illustrated in Figure 3ci. The width of these hydrophilic channels (*L*) can be precisely controlled by adjusting the width of the template (*W*_T_) between the two adjacent strips in the mask. Figure 3cii shows the formation of hydrophilic channels in various sizes, corresponding to different *W*_T_ values ranging from 0.5 to 2.5 mm (detailed microscopic images are shown in Figure S11). The *W*_F_ and *W*_B_ values of the hydrophobic barrier are constant at around 2 mm due to the fixed width of the exposed area (2 mm in width) on the mask (Figure 3ciii). The widths of the front and back of the hydrophilic channel (*L*_F_ and *L*_B_) increase with increasing *W*_T_, as described by the following equations:

$$L_{F}=0.98\times W_{T}-0.034(R^{2}\;\mathrm{of}\;0.999)$$

$$L_{B}=0.95\times W_{T}-0.085(R^{2}\;\mathrm{of}\;0.998)$$

With a *W*_T_ value of 1 mm, the resulting *L*_F_ and *L*_B_ values for the hydrophilic channel are 0.95 and 0.87 mm, respectively. The slightly lower *L*_F_ and *L*_B_ values than that of the designed *W*_T_ are attributed to the cumulative horizontal spreading of the two adjacent Parafilm barriers. The fact that the *L*_F_ value is greater than *L*_B_ again implies that the horizontal spreading of the molten Parafilm is more restricted on the paper-front side than on the paper-back side. Ultimately, the hot-pressing technique enables the resolution of these hydrophilic channels to reach the submillimeter range, achieving a minimum resolution of approximately 0.45 mm using a mask with a *W*_T_ of 0.5 mm (Figures 3ciii and S11). Since the sizes of channels and zones in μPAD devices are usually on the order of 1–5 mm,^30^ this method provides sufficient resolution for effective fabrication of μPAD devices on a paper substrate.

The patterned paper-para was subjected to wettability measurements, SEM imaging, and fluidic characterization. Figure 4a demonstrates the wettability of the original paper, Parafilm, and paper-para as measured by the static water contact angle (WCA, θ). The original paper exhibited immediate wetting (i, 0.2 s) upon contact with a water droplet, attributed to its hydrophilicity, with the droplet dissipating after approximately 1.3 s (ii) owing to capillary wicking action (a consecutive record of the droplet on the paper surface can be seen in Figure S12). Parafilm, being hydrophobic, displays a WCA value of 106.9 ± 1.8°. After hot-press patterning, the paper-para shows similar hydrophobicity, with WCA values of 110.2 ± 2.3° and 113.43 ± 3.2° for paper-para-front and paper-para-back, respectively. Figure 4b presents a cross-sectional SEM image of paper-para after hot-press patterning, showing multilayers of cellulose fibers on the left (Paper) and a Parafilm coating on the right (Paper-para). The Parafilm coating protrudes about 90 μm above the paper-front surface, with a diffusion boundary observed between the paper and paper-para (the protruding and diffusive effects are schematically illustrated and explained in Figure S13). The high-magnification image (ii) indicates the coating on the paper-front and its infusion through the paper to the paper-back. The top-view SEM image in Figure 4c displays a clear and well-defined fluidic channel (highlighted by the red dashed lines) between the paper-front and paper-para-front with a protruding boundary. No protruding effect of Parafilm can be observed at the paper-para-back (Figure 4d), which also shows a well-defined channel and consistent coverage, similar to the paper-para-front.

**Figure 4:**
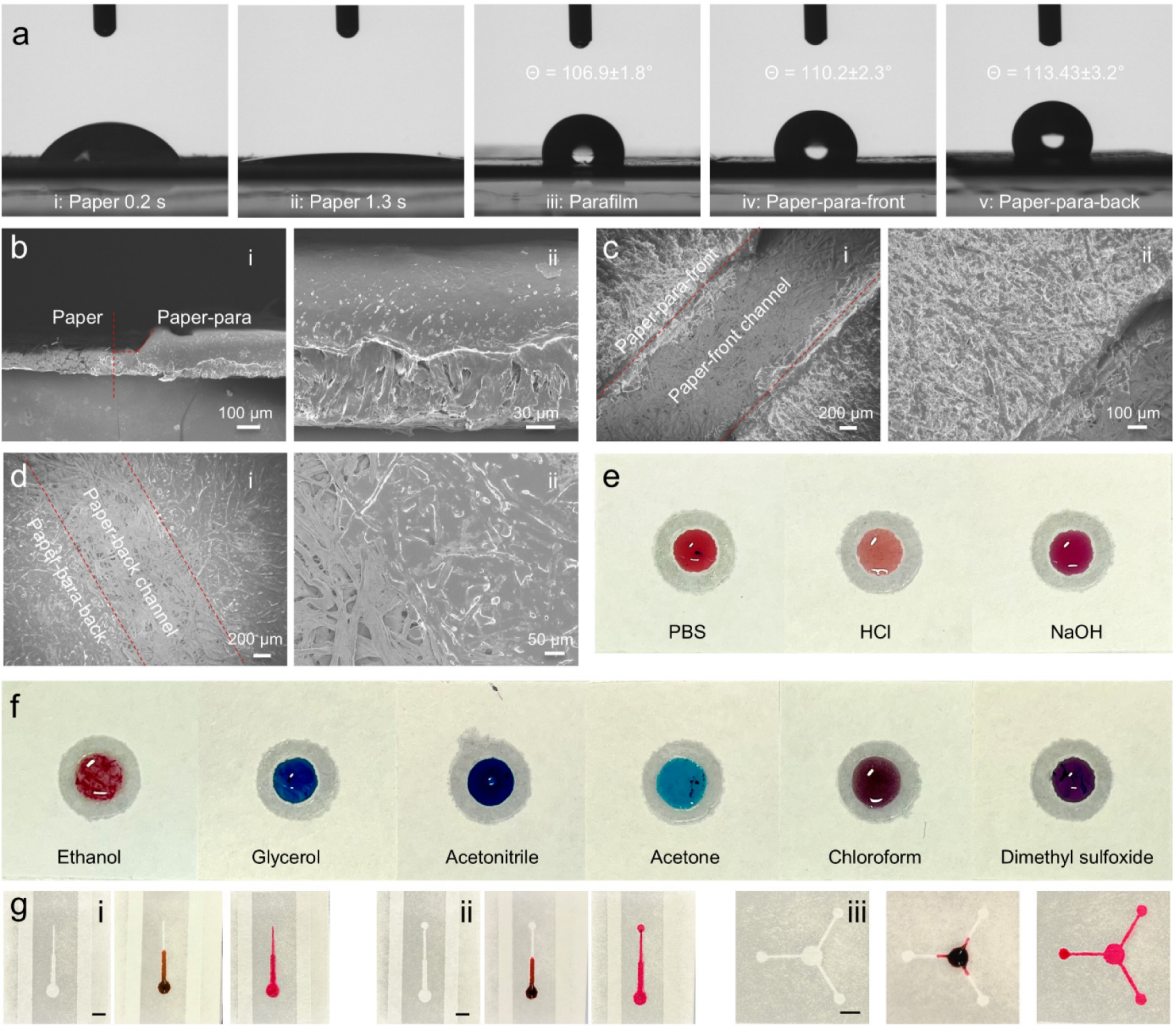
Paper-para properties. (a) Wettability measurements for (i, ii) paper, (iii) Parafilm, (iv) paper-para-front, and (v) paper-para-back (*n* = 5). (b) Cross-sectional SEM images of paper-para at two magnifications (i) and (ii). The red dashed lines in (i) indicate the protruding and diffusive effects of Parafilm as well as the boundary between paper and paper-para. (c) Top-view SEM image of the paper-para-front at two magnifications (i) and (ii). The red dashed line indicates the boundary between the paper-front strip and the paper-para-front. (d) Top-view SEM image of the paper-para-back with a high magnification (di), red dashed line indicates the boundary between the paper-back strip and the paper-para-back. Solvent compatibility tests using a variety of aqueous solutions (e) and organic solvents (f) dropped in the circular region confined by paper-para; various types of dyes were added to the solutions and solvents for visualization. (g) Examples of paper microfluidic patterns with a red dye solution flowing through (i) a single fluidic channel connected to a sampling zone at one end, (ii) a single fluidic channel connected to a sampling zone and a detection zone at two ends, and (iii) triple-fluidic-channels connected to a central sampling zone and three detection zones, the scale bar is 5 mm.

The solvent compatibility of the hot-press patterned paper was assessed using a variety of aqueous solutions and organic solvents. As shown in Figure 4e, droplets of aqueous solutions with various pH values, including PBS (0.1 M) at a neutral pH of 7, hydrochloric acid solution (1 M), and sodium hydroxide solution (1 M), were well confined within the circular hydrophilic paper region (diameter of 5 mm), bordered by hydrophobic paper-para. Beyond aqueous solutions, the paper-para successfully retained all tested organic solvents within the central circular region (Figure 4f), including ethanol, glycerol, acetonitrile, acetone, chloroform, and dimethyl sulfoxide. After resting at an ambient temperature for 1 h, the circular region remained intact, with no disruption to the paper-para barriers (Figure S14). These results indicate that the patterned paper-para exhibits superior compatibility and stability across a wide range of solutions and solvents, outperforming those produced by wax printing, laser printing, and thermal transfer printing.^29,30,33^ Various paper microfluidic patterns were achieved with a rapid flow of red dye solution through the designed channels, as shown in Figure 4g, including (i) a single fluidic channel connected to a sampling zone at one end, (ii) a single fluidic channel connected to a sampling zone and a detection zone at two ends, and (iii) triple fluidic channels connected to a central sampling zone and three detection zones.

### 3.3 Integrating PLIG with Microfluidics on a Single Monolithic Paper Sheet for μPLIG

In paper-based electroanalytical devices, fluidic patterning is crucial not only for guiding and manipulating fluidic flows on the paper platform but also for protecting electroactive regions (e.g., conductive tracks and contact pads) by blocking unwanted fluidic interactions. Therefore, to develop integrated papertronic components with microfluidics on a single sheet of paper, the compatibility of PLIG with hot-pressing was first evaluated using a typical 3-electrode system as a model. The 3-electrode system remained intact postpressing (Figure S15), while leaving black-colored PLIG residues on the mask. This observation is consistent with our previous findings for LIG derived from polyimide.^12^ Figure 5a displays SEM images of the PLIG after hot-pressing, showing no significant alterations in the fibrous and graphitic structure (i), though the high-magnification image (ii) reveals the removal of graphitic snippet residues from the edge of the fibers compared to PLIG before pressing (Figure 2av). This indicates that the residue on the mask is caused by peeling off graphitic snippets from the PLIG fiber edge. Electrochemical performance of the 3-electrode PLIG after hot-pressing was assessed by CV (Figure 5b) and EIS (Figure 5c). The Ipa value of PLIG after hot-pressing is 100.3 μA, which is slightly lower than that of the original PLIG-DeFo. Additionally, the Δ*E* value increased to 165 mV (from 149 mV), and the *R*_ct_ increased to 190.8 Ω (from 122.4 Ω). These results indicate that the hot-pressing process impacts the electrochemical performance to some extent; however, PLIG after hot-pressing still outperforms PLIG-De and PLIG-Fo before hot-pressing (Figure 2d,e), demonstrating an acceptable compatibility of hot-press patterning with PLIG. This is crucial for integrating paper electronics with microfluidics, ensuring well-defined flow channels and detection zones across conductive patterns.

**Figure 5:**
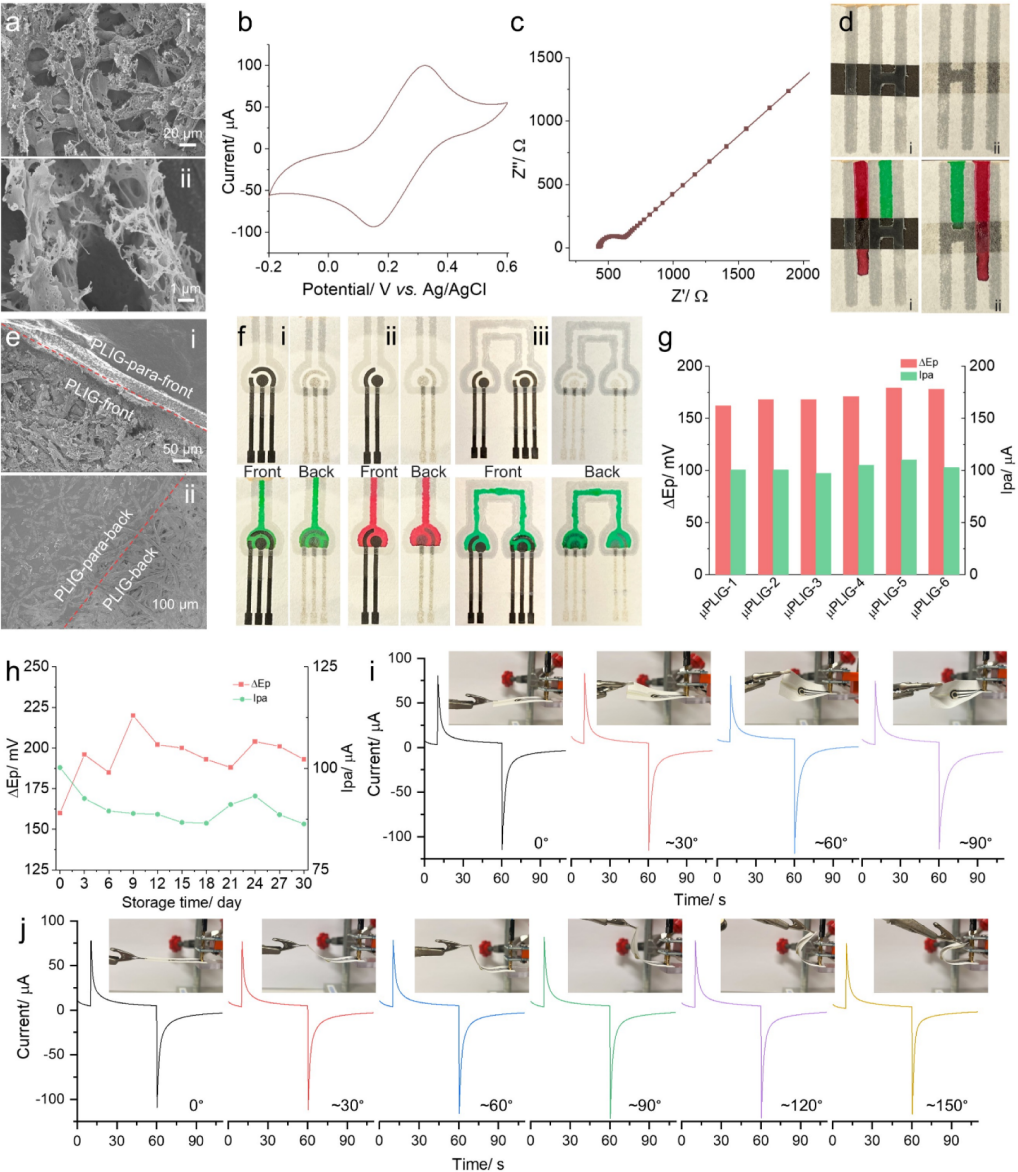
Integration of microfluidics with PLIG for μPLIG. (a) SEM images of PLIG after hot-pressing in low (i) and high (ii) magnifications. (b) CV and (c) EIS measurements of the 3-electrode PLIG after hot-pressing in 5 mM Fe­(CN)_6_^3–/4–^ in 0.1 M KCl. (d) Hydrophilic channels on PLIG (i: front side; ii: back side) defined by hydrophobic Parafilm. Flow of dye solutions (bottom) where the flow channel of the green solution is blocked in the middle of the images. (e) SEM images of Parafilm-infused PLIG (PLIG-para) in the front (i) and back (ii) views, where the red dashed line indicates the boundary between the PLIG-para and PLIG. (f) Digital photograph of μPLIG with a flow channel and a detection zone onto a 3- (i), 2-(ii), and dual 3-/2- (iii) electrode systems, with dye solution indicating the fluidic flow. (g) Reproducibility of the 3-electrode μPLIG. (h) Storage stability of the 3-electrode μPLIG under ambient conditions over 30 days. Current–time curve for twisting (i) and bending (j) effects on the electrochemical performance of the 3-electrode μPLIG.

In addition to the compatibility investigation of PLIG with the hot-pressing technique, the definition of hydrophilic channels by hydrophobic Parafilm across conductive PLIG is demonstrated in Figure 5d. Two flow channels (3 mm wide) were patterned perpendicular to a PLIG strip (5 mm), with one channel blocked in the middle of the PLIG strip by Parafilm. The red dye solution (channel not blocked) flowed across the PLIG strip, indicating the retention of capillary flow capacity after generating PLIG on the cellulose paper substrate. Conversely, the flow of the green dye solution (channel blocked) was halted in the middle of the PLIG strip due to the hydrophobic Parafilm pattern (visualized in Video S1). Figure 5e shows a clear boundary (highlighted by the red dashed line) between the Parafilm-infused PLIG-front (PLIG-para-front) and the PLIG-front (i), as well as the successful infusion of Parafilm throughout the PLIG, with a clear boundary between the PLIG-para-back and the PLIG-back (ii). To demonstrate the feasibility of integrating PLIG with microfluidics, we patterned fluidic channels and detection zones onto the PLIG platform (Figure 5f, top) with standard 3- (i), 2- (ii), and dual 3-/2- (iii) electrode system configurations. The flow along the channels to the detection zones was visualized by green/red dye solutions on both front and back side views (Figure 5f, bottom; corresponding flow videos can be seen in Videos S2–S4).

The reproducibility of the μPLIG fabrication process was assessed by analyzing the Ipa and Δ*E* values obtained from CV using six 3-electrode μPLIGs, as shown in Figure 5g (corresponding CV curves are provided in Figure S16). The average Ipa and Δ*E* values were 102.6 μA and 170.3 mV, with relatively low relative standard deviations (RSDs) of 4.5% and 5.7%, respectively, indicating good and seamless integration reproducibility of μPLIG from PLIG and hot-press microfluidic patterning. The storage stability of μPLIG was evaluated by monitoring changes in Ipa and Δ*E* values over 30 days of storage under ambient conditions using the same batch of μPLIGs. As shown in Figure 5h, after 3 days of storage, the Ipa value decreased to ∼90% of the freshly prepared μPLIG, while the Δ*E* value increased to 196 mV. Such a decrease in electrochemical performance upon storage for LIG was ascribed to the adsorption of adventitious hydrocarbons from the storage environment, as reported in previous reports.^47^ Then, the μPLIG remained relatively stable during further storage, maintaining an Ipa value of 88.5 ± 2.5 μA and a Δ*E* value of 198.4 ± 9.8 mV (corresponding CV curves are provided in Figure S17). The effect of mechanical deformation on the μPLIG platform was evaluated, considering its inherent flexibility. Figure 5i,j shows the multistep current responses (equilibration, oxidation, and reduction) under different twisting and bending angles, respectively. Detailed plots of current values versus twisting/bending angles are provided in Figure S18. Insignificant variations in the equilibration, oxidation, and reduction current values were observed across various twisting/bending angles, indicating both good flexibility and mechanical stability.

The μPLIG offers advantages in fabrication simplicity and material sustainability. Unlike previously reported LIG-based microfluidic devices that rely on synthetic polymer substrates (i.e., polyimide) and require multimaterial assembly involving PDMS, PMMA, glass/acrylic, or 3D-printed housings,^16–19^ our approach achieves seamless integration of both sensing and fluidic functions on a single sheet of paper. This eliminates the need for external enclosures or structural support components, significantly simplifying the device architecture and supporting full eco-disposability as well as flexibility. A detailed comparison with representative LIG-microfluidic platforms is provided in Table S3.

### 3.4 Demonstrative Aqueous and Organic Sensing Applications

The integrated μPLIG with the 3- and 2-electrode systems was employed for potentiometric pH sensing and amperometric lactate biosensing in aqueous systems by injecting 10 μL of samples at the terminal of the channel. As depicted in Figure 6a, the functionalized 2-electrode μPLIG responded to various pH values, with the potential gradually decreasing from around 50 mV to −70 mV as the pH value increased from 6 to 9. The corresponding calibration curve demonstrates a linear relationship between potential and pH value, with a sensitivity of −40.3 mV pH^–1^. For lactate biosensing (Figure 6b), the amperometric curve exhibited a distinct current response to a dynamic range of lactate concentrations up to 7.5 mM, with a sensitivity of 0.92 μA mM^–1^. Beyond the sensing applications in aqueous systems, the integrated μPLIG is also promising for detection of organic compounds in organic systems, leveraging the good compatibility of the μPLIG electroanalytical platform toward various types of organic solvents. As shown in Figure 6c, the voltammetric curve showed an increased trend in the electrooxidation peak current with increased concentration of Vitamin D3 in an ethanol mixture. The linear range was 5–65 μM, with a limit of detection (LOD) of 1.32 μM. The electroanalytical performance of the μPLIG platform toward pH sensing, lactate biosensing, and Vitamin D3 detection was comparable with reported values in the literature (Table S4). These demonstrations show the compatibility and versatility of the developed μPLIG as an electroanalytical platform in both aqueous and organic systems. It is also promising to design and combine more functions conferred by paper microfluidics, such as sample pretreatment and prereaction (e.g., incubation), to achieve a fully integrated, reagentless, and sustainable electroanalytical platform for a wide range of applications.

**Figure 6:**
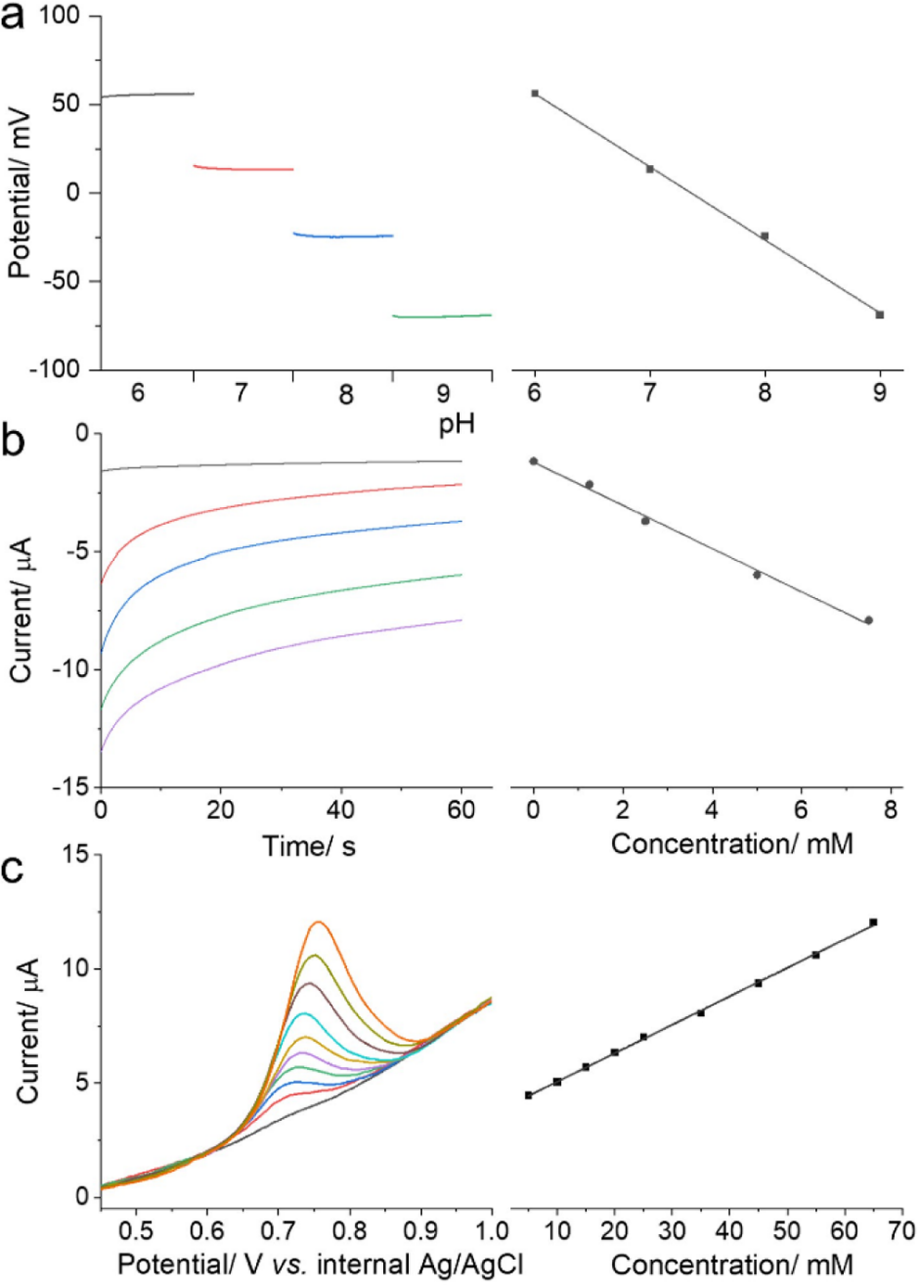
(a) Potentiometric pH sensing over a pH range of 6–9 at a functionalized 2-electrode μPLIG platform, with the potentiometric response curve (left) and the corresponding calibration curve of the potential versus pH values (right). (b) Amperometric lactate biosensing over a lactate range of 0–7.5 mM at a functionalized 3-electrode μPLIG platform, with the amperometric response curve (left) and the corresponding calibration curve of the current versus lactate concentration (right). (c) Voltammetric Vitamin D3 sensing over a range of 5–65 μM at the 3-electrode μPLIG platform, with the DPV response curves (left) and the corresponding calibration curve of oxidation peak current versus Vitamin D3 concentration (right).

## 4 Conclusions

In summary, we have demonstrated a seamless integration of laser-induced papertronics and Parafilm-based microfluidics on a single monolithic paper sheet as a versatile electroanalytical platform in both organic and aqueous systems. By employing borax and laser treatment, we directly converted cellulose paper into laser-induced graphite, enabling the creation of customized conductive pathways and electronic components. Microfluidic channels were patterned through a hot-pressing technique using hydrophobic Parafilm, allowing for submillimeter resolution patterning at a relatively low temperature and short processing time. Compared to conventional wax-patterned μPADs and existing microfluidic LIG devices, the seamless integration of PLIG with the microfluidics (μPLIG) platform introduces innovations of enhanced chemical compatibility with a broad range of aqueous and organic solvents and monolithic integration of sensing and fluidic elements on a single biodegradable substrate without adhesives and multilayer assemblies. The resulting μPLIG platform demonstrates compatibility with both aqueous and organic solvents, enabling the scope of electroanalytical applications from pH sensing and lactate biosensing in aqueous solutions to organic compound detection in organic solvent systems. This study paves the way for developing a cost-effective, disposable, and versatile electroanalytical platform for on-site analysis. Beyond the current reliance on incineration for the disposal of paper-based electronics,^7,48,49^ future efforts could explore the use of eco-friendly and biodegradable materials for paper patterning and functionalization to achieve a fully sustainable and disposable platform, as well as extend the validation of μPLIG-based sensing to complex real-world samples to further establish its analytical utility.

## Supplementary Material











## Notes

Sheet resistance measurement of PLIG at various laser scan speeds and laser power; optical microscopic images of PLIG at various laser scan speeds and laser power; SEM images of PLIGS10P22.5; FTIR, EDX, XPS full survey, and XPS O 1s/C 1s of various paper and PLIG; summary of element and component percentages from XPS; summary of electrochemical performance; optical images of paper-para and its solvent compatibility test; PLIG conductivity calculation; consecutive recordings of water droplets on paper; schematic illustration of the diffusion and protruding effect of Parafilm on the paper; solvent compatibility test; digital photograph of 3-electrode PLIG after hot-pressing; CV curves of μPLIG; plots of multistep current values (equilibration, oxidation peak and reduction peak) upon bending and twisting; comparison of the μPLIG with reported microfluidic LIG; comparison of the electroanalytical performance of the μPLIG platform with the literature (PDF)
Flow of the red/green dye solution alongside the unblocked and blocked channels, respectively (MP4)
Flow of the dye solution along the channel to a detection zone in a 3-μPLIG system (MP4)
Flow of the dye solution along the channel to a detection zone in a 2-μPLIG system (MP4)
Flow of dye solution along the channel to a detection zone in a dual 3-/2-μPLIG system (MP4)
