# Supplementary material for: Seamless Integration of Laser-Induced Papertronics with Parafilm-Based Microfluidics as a Versatile Paper-Based Electroanalytical Platform
Source: ACS Appl Mater Interfaces. 2025 Jun 26;17(27):39719–31. doi: 10.1021/acsami.5c09316 (PMC12257460; doi:10.1021/acsami.5c09316)
Supplement: Supplementary file 1 [file am5c09316_si_001.pdf]

Supporting Information

Seamless Integration of Laser-induced Papertronics with Parafilm-based Microfluidics as a Versatile Paper-based Electroanalytical Platform

Lingyin Meng[a], Danfeng Cao[b], Jonas Oshaug Pedersen[c], Grzegorz Greczynski[d], Vladyslav Rogoz[d], Warakorn Limbut[e], Mats Eriksson[a]*

[a]Division of Sensor and Actuator Systems, Department of Physics, Chemistry and Biology, Linköping University, 581 83, Linköping, Sweden

[b]Laboratory of Organic Electronics, Department of Science and Technology, Linköping University, 601 74 Norrköping, Sweden

[c]Division of Biophysics and Bioengineering, IFM, Linköping University, 581 83 Linköping, Sweden

[d]Thin Film Physics Division, Department of Physics, Chemistry and Biology (IFM), Linköping University, SE-581 83 Linköping, Sweden

[e]Center of Excellence for Trace Analysis and Biosensor, Prince of Songkla University, Hat Yai, Songkhla, 90110, Thailand

*Corresponding Author

Email: lingyin.meng@liu.se

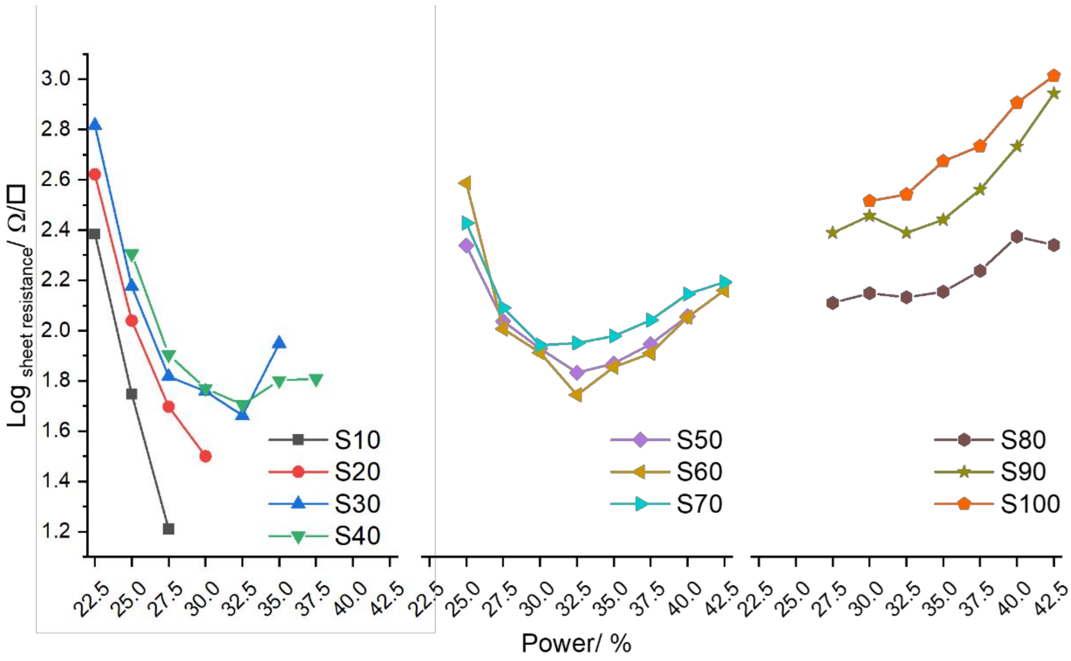

Figure S1. Sheet resistance of PLIG as a function of laser scan speed (S10–100) and laser scan power (P22.5–42.5).

The laser power and scan speed are critical factors influencing the graphitization yield and thus the electrical properties of the PLIG. As shown in Figure S1, the sheet resistance of the PLIG fabricated under defocus conductions was evaluated across varied laser power (P20–42.5%) and scan speed (S10–100). Representative optical microscopic images of the resulting PLIG generated under these conditions are displayed in Figure S2a–j. For a scan speed of S10, P20 was not effective for converting cellulose into PLIG. As the laser power increased from P22.5 to P27.5, the sheet resistance value (logarithmic-scale, *Log*, base 10) of the resulting PLIG decreased from 2.38 to 1.21. Further increase of power led to burn-through of the paper substrate (Figure S2a). Higher scan speed (S20–100), which corresponds to decreased laser exposure time, resulted in a higher sheet resistance value, under which circumstance, a high laser power is needed for good conductivity. It is worth noting that an inflection point appeared when the scan speed exceeded S30, and an increased sheet resistance was observed for increased laser power. This can be attributed to the evident burn-through holes in the PLIG and their detriment to the conductance. S10P25 was chosen for PLIG fabrication since this setting resulted in the best compromise between conductivity and PLIG mechanical properties (adhesion, no burn-through).

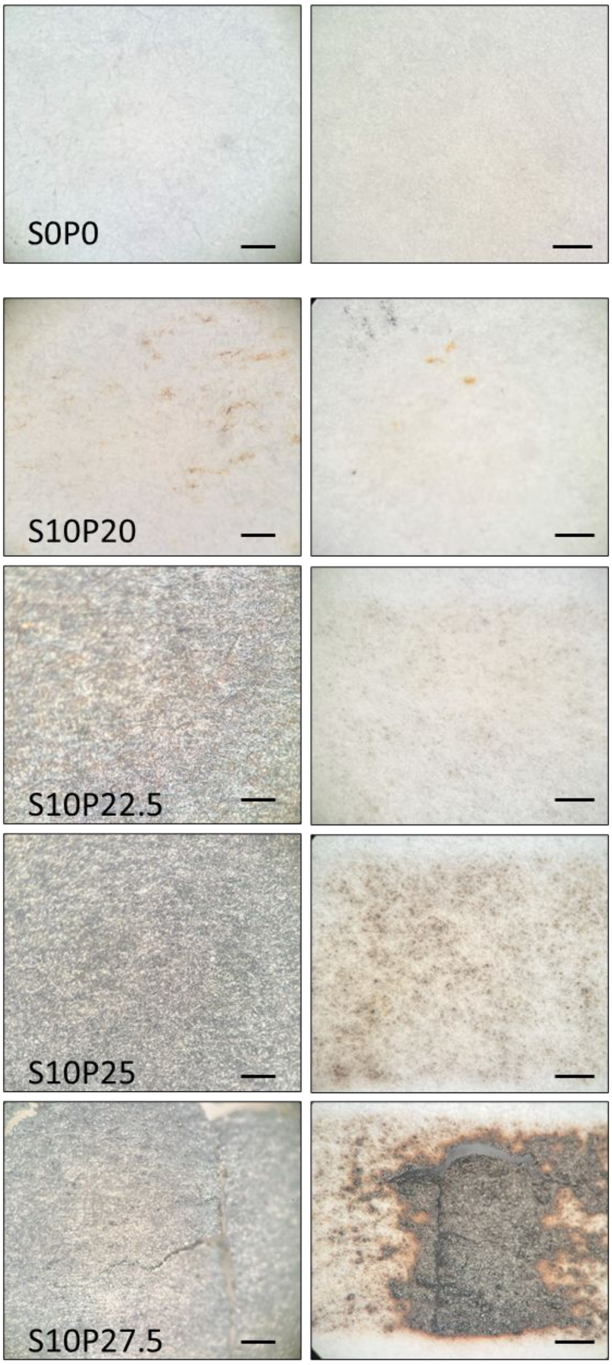

Figure S2a. Optical microscopic image (left: front view in scale bar of 0.3 mm, right: back view in scale bar of 1 mm) of paper (S0P0) and PLIG with 10% of the laser speed (S10) and various percent of power (P20–27.5).

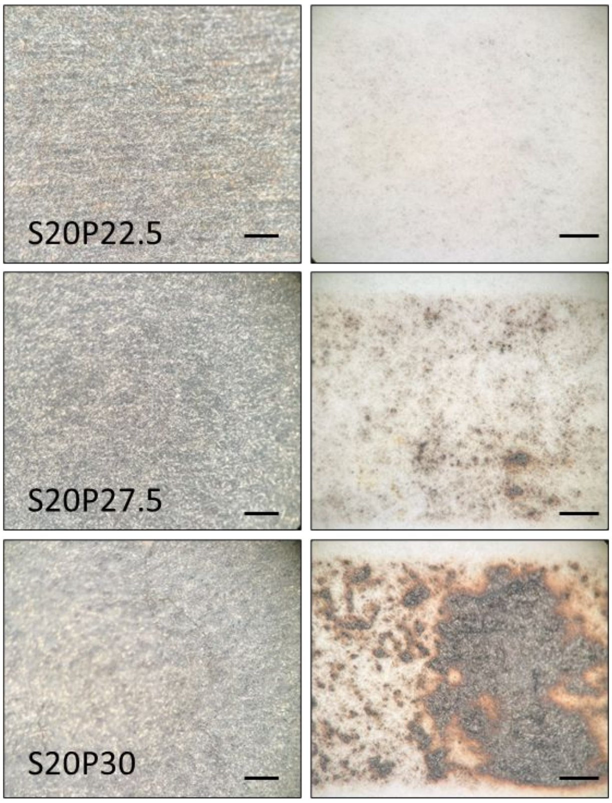

Figure S2b. Optical microscopic image (left: front view in scale bar of 0.3 mm, right: back view in scale bar of 1 mm) of PLIG with 20% of the laser speed (S20) and various percent of power (P22.5–30).

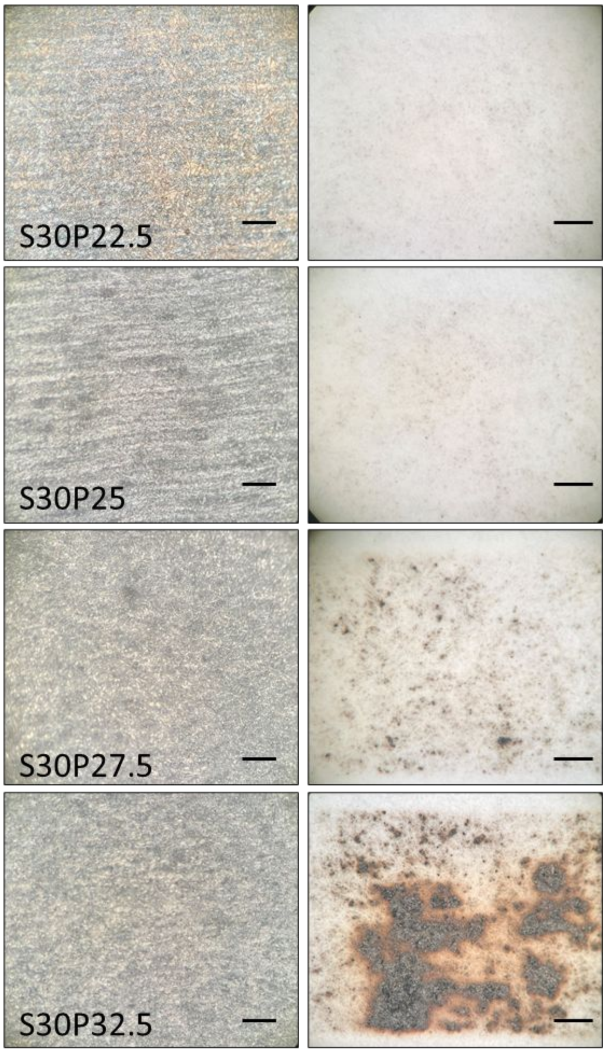

Figure S2c. Optical microscopic image (left: front view in scale bar of 0.3 mm, right: back view in scale bar of 1 mm) of PLIG with 30% of the laser speed (S30) and various percent of power (P22.5–32.5).

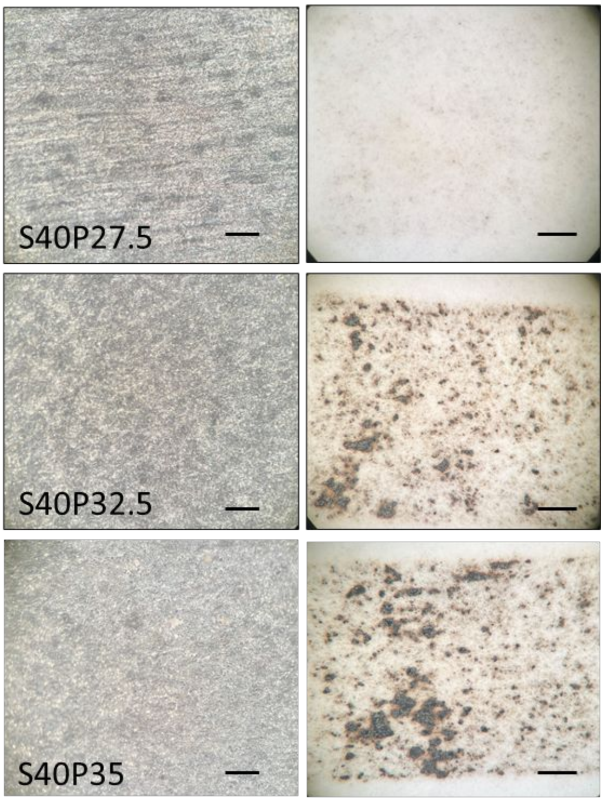

Figure S2d. Optical microscopic image (left: front view in scale bar of 0.3 mm, right: back view in scale bar of 1 mm) of PLIG with 40% of the laser speed (S40) and various percent of power (P27.5–35).

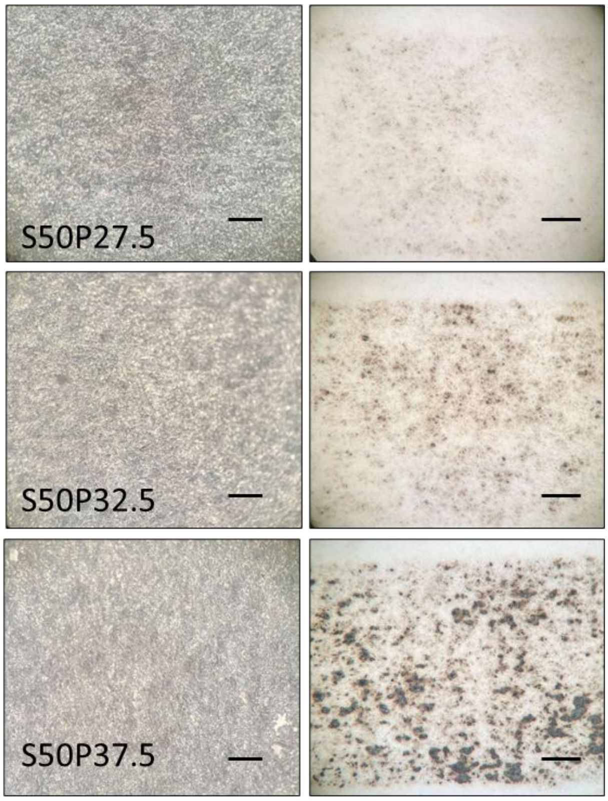

Figure S2e. Optical microscopic image (left: front view in scale bar of 0.3 mm, right: back view in scale bar of 1 mm) of PLIG with 50% of the laser speed (S50) and various percent of power (P27.5–37.5).

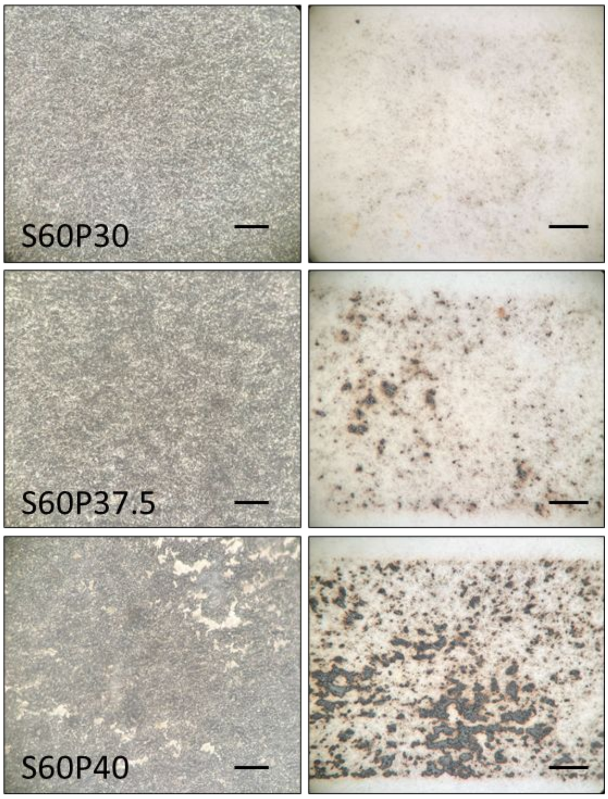

Figure S2f. Optical microscopic image (left: front view in scale bar of 0.3 mm, right: back view in scale bar of 1 mm) of PLIG with 60% of the laser speed (S60) and various percent of power (P30–40).

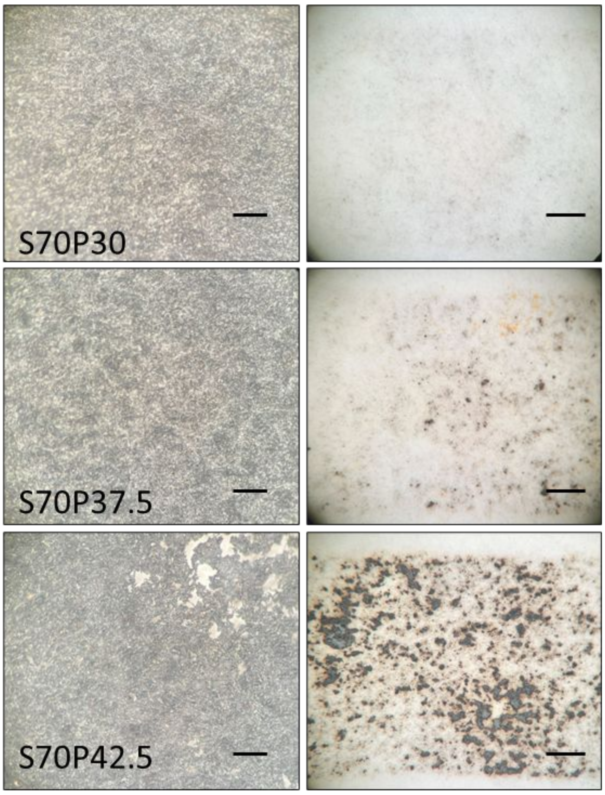

Figure S2g. Optical microscopic image (left: front view in scale bar of 0.3 mm, right: back view in scale bar of 1 mm) of PLIG with70% of the laser speed (S70) and various percent of power (P30–42.5).

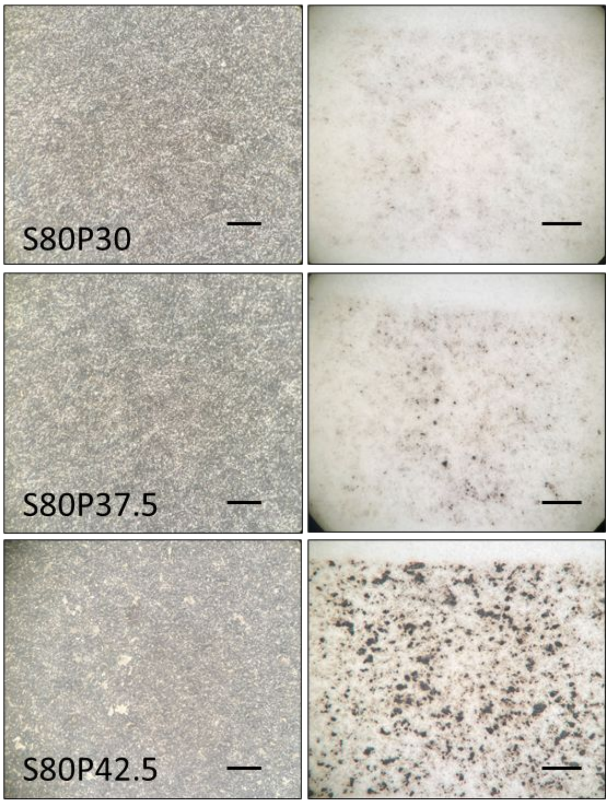

Figure S2h. Optical microscopic image (left: front view in scale bar of 0.3 mm, right: back view in scale bar of 1 mm) of PLIG with 80% of the laser speed (S80) and various percent of power (P30–42.5).

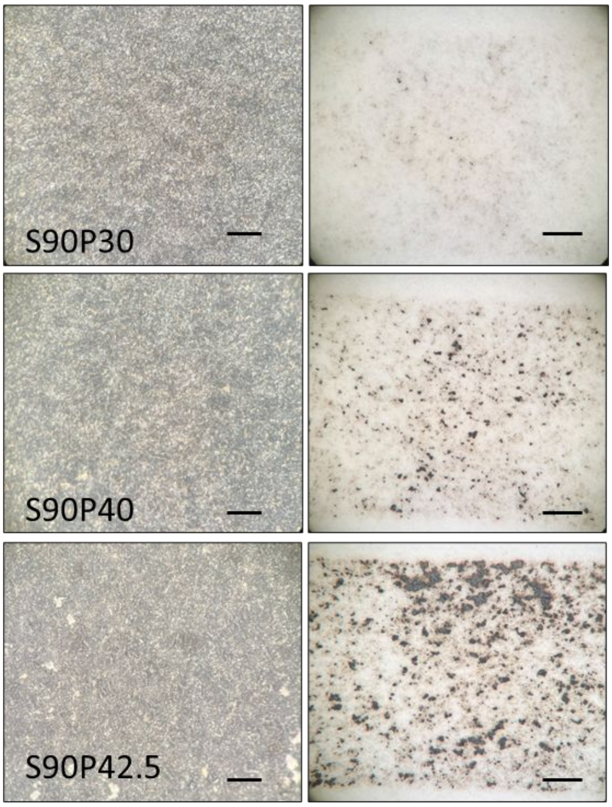

Figure S2i. Optical microscopic image (left: front view in scale bar of 0.3 mm, right: back view in scale bar of 1 mm) of PLIG with 90% of the laser speed (S90) and various percent of power (P30–42.5).

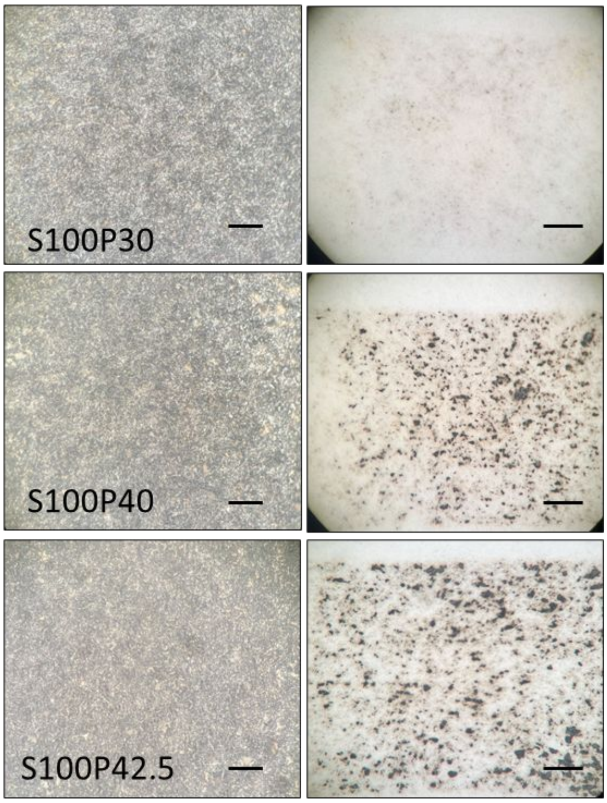

Figure S2j. Optical microscopic image (left: front view in scale bar of 0.3 mm, right: back view in scale bar of 1 mm) of PLIG with 100% of the laser speed (S100) and various percent of power (P30–42.5).

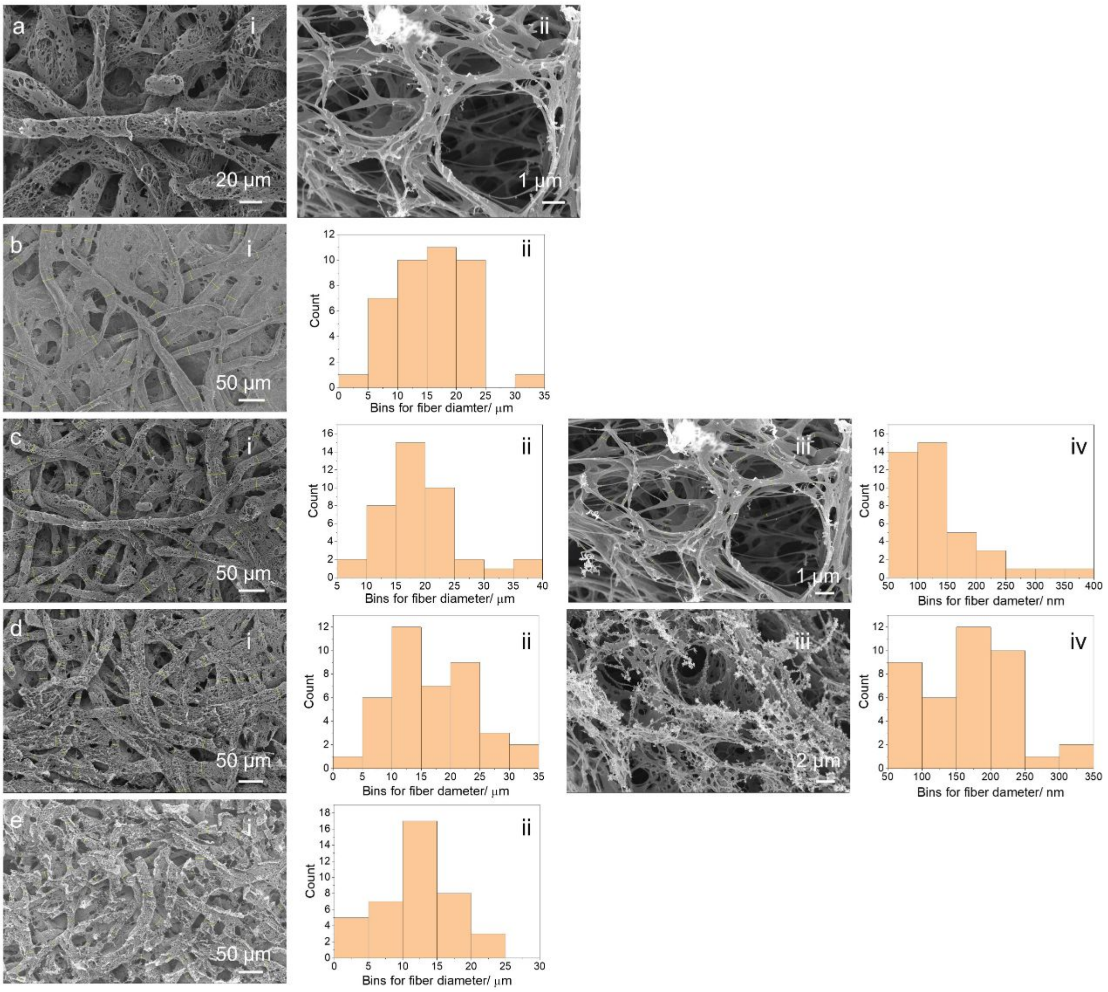

Figure S3. (a) SEM images, at two different magnifications of PLIG produced under the laser treatment of S10P22.5 in defocus mode. (b) Original cellulose, SEM image (i) and corresponding diameter distribution histogram (ii) of counts versus bin center of fibre diameter. (c) PLIG-De at S10P22.5, SEM images (i, iii) and corresponding microfiber (ii) and nanofiber (iv) diameter distribution histograms. (d) PLIG-De at S10P25, SEM images (i, iii) and corresponding microfiber (ii) and nanofiber (iv) diameter distribution histograms. (e) PLIG-DeFo, SEM image (i) and corresponding microfiber (ii) diameter distribution histogram. All fiber diameters were manually measured as image pixels using Image J processing software then correlated to a pre-determined pixel-to-length scale based on the SEM scale bar, based on the reported approach in literature[1]. 40 measurements were conducted for each SEM image, which were marked with yellow dash lines.

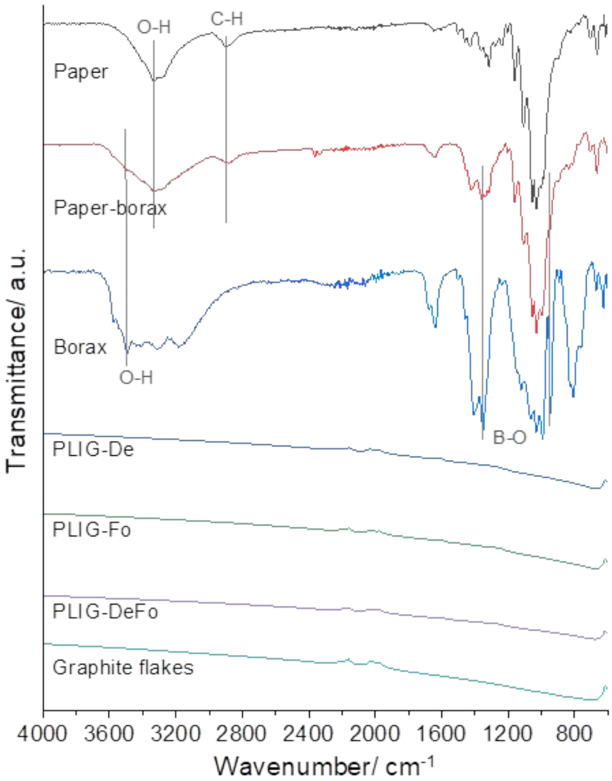

Figure S4. FTIR of paper, paper-borax, borax, PLIG-De, PLIG-Fo, PLIG-DeFo, and graphite flakes as a control sample.

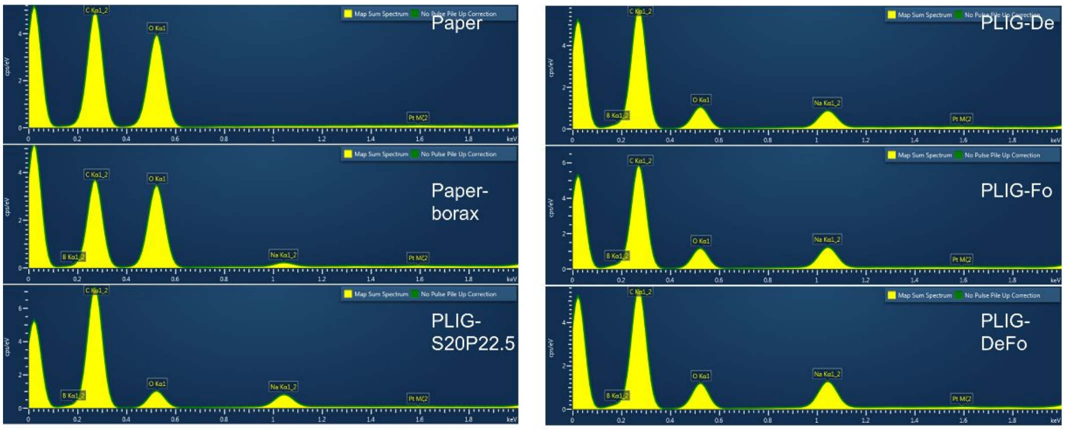

	C	O	B	Na
Paper	58.30	41.70		
Paper-borax	58.33	35.68	5.69	0.30
PLIG-S20P22.5	78.38	13.62	7.17	0.83
PLIG-De	77.90	12.56	8.52	1.02
PLIG-Fo	76.79	12.81	9.08	1.32
PLIG-DeFo	76.23	13.62	8.69	1.46

Figure S5. EDX spectra of paper, paper-borax, PLIG-S20P22.5, PLIG-De, PLIG-Fo, PLIG-DeFo, and the chemical composition summary in atomic percentage.

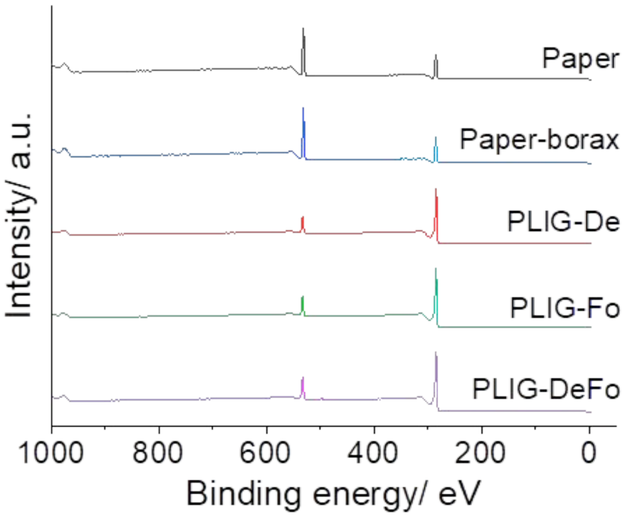

Figure S6. XPS full survey spectra of paper, paper-borax, PLIG-De, PLIG-Fo, and PLIG-DeFo.

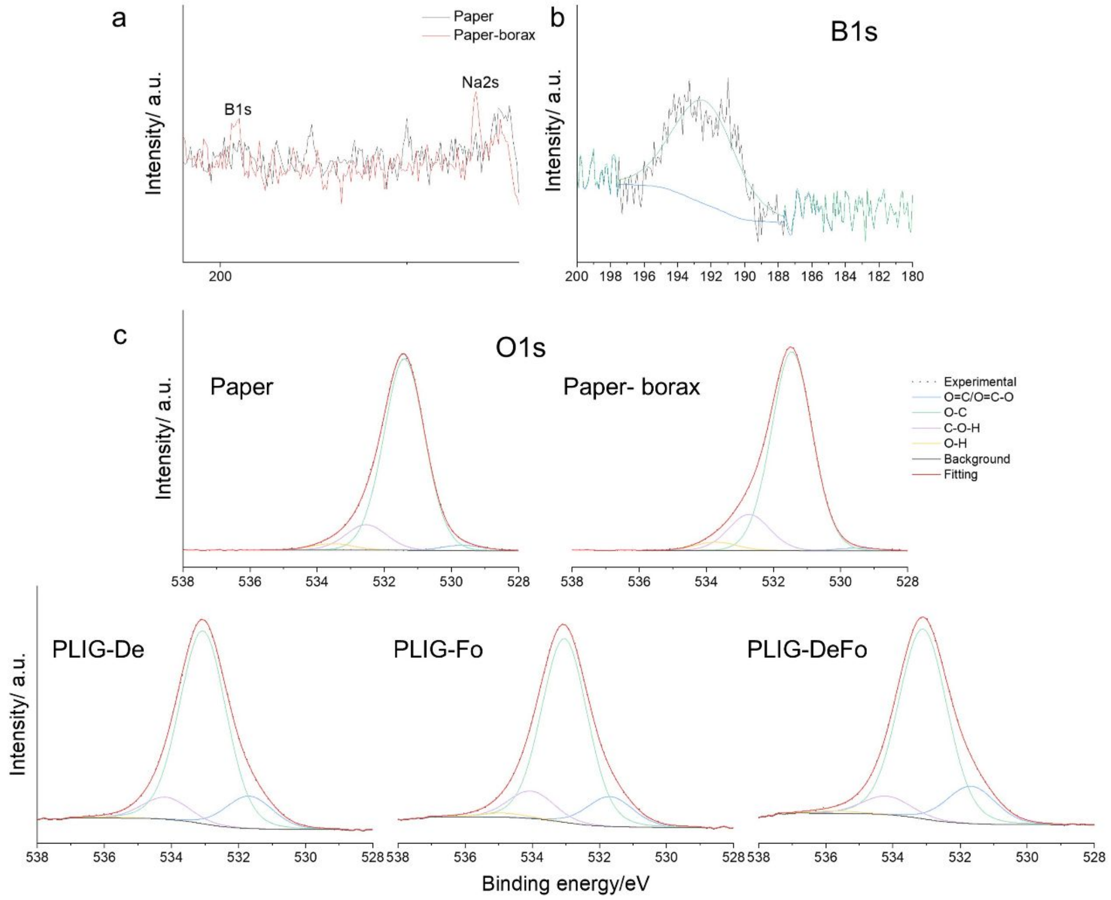

Figure S7. XPS spectra. (a) Magnified region of full survey for paper and paper-borax. (b) B1s of paper-borax. (c) O1s spectra of paper, paper-borax, PLIG-De, PLIG-Fo, and PLIG-DeFo.

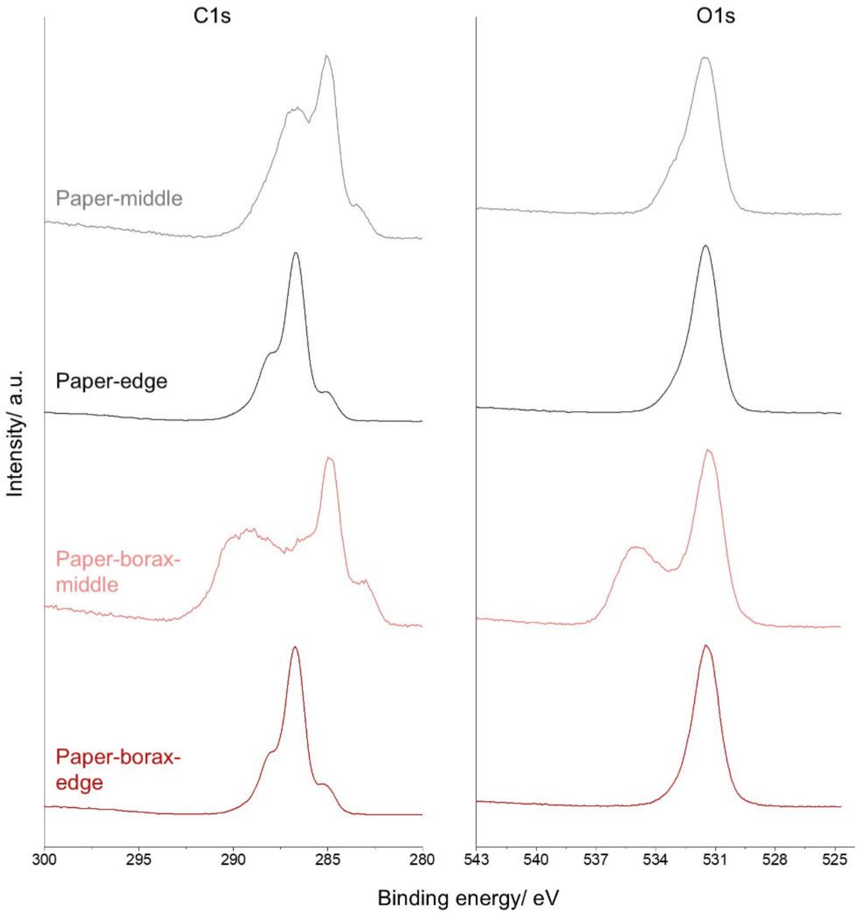

Figure S8. XPS C1s (left) and O1s spectra for paper and paper-borax. The spectra are presented from top to bottom as follows: cellulose paper (middle spot, Paper-middle), cellulose paper (edge spot, Paper-edge), cellulose paper-borax (middle spot, Paper-borax-middle), and cellulose paper-borax (edge spot, Paper-borax-edge).

The XPS spectra were obtained by measuring two different spots at the paper and paper-borax, including middle spot (located in the middle of the sample) and edge spot (located at the sample edge, close to the metal contact of Au/Si substrate), due to their non-conducting properties. As shown in Figure S8, the measurements at the middle spot exhibited more significant binding energy shifts and deterioration in the high binding energy region in both the C1s and O1s spectra, for both paper and paper-borax samples. As noted in previous reports, deterioration in spectral resolution at the high binding energy region of the C1s signal is a typical indicator of inhomogeneous charging artifacts often encountered in XPS analysis of cellulose-based materials[2, 3]. In contrast, the spectra obtained from the edge spot showed improved spectral resolution, likely due to reduced charging effects.

Table S1. Summary of element and component percentages.

	Survey				C1s					O1s			
	C	O	B	Na	C-C	C-O	O-C-O	C=O	O-C=O	C=O	O-C	C-O-H	O-H/O-B
Paper	62.73	37.27	-	-	9.86	66.69	20.89	2.55	-	2.33	83.83	11.10	2.74
Paper-borax	61.15	37.85	0.58	0.42	10.73	69.02	18.56	1.69	-	1.04	80.93	14.66	3.37
PLIG-De	90.56	9.44	-	-	73.39	12.64	5.54	6.64	1.79	13.09	77.78	8.86	0.27
PLIG-Fo	91.25	8.75	-	-	74.63	12.29	5.08	6.33	1.67	12.20	74.92	11.19	1.69
PLIG-DeFo	90.18	9.82	-	-	73.84	12.50	5.26	6.54	1.86	11.80	79.02	8.35	0.83

Table S2. Summary of anodic peak current and peak-to-peak separation (ΔE) from CV curves and EIS fitting values.

Sample	CV		EIS				
	Ipa/ μA	ΔE/ mV	Rs/ Ω	Rct/ Ω	CPE/ $\mu s^{\alpha}/\Omega$	α	W/ Ω/√s
PLIG-DeFo	107.4	149	474.9	122.4	11.2	0.85	615.5
PLIG-De	87.3	257	325.1	413.7	3.44	0.90	753.1
PLIG-Fo	92.3	245	468.7	468.0	11.45	0.88	753.7

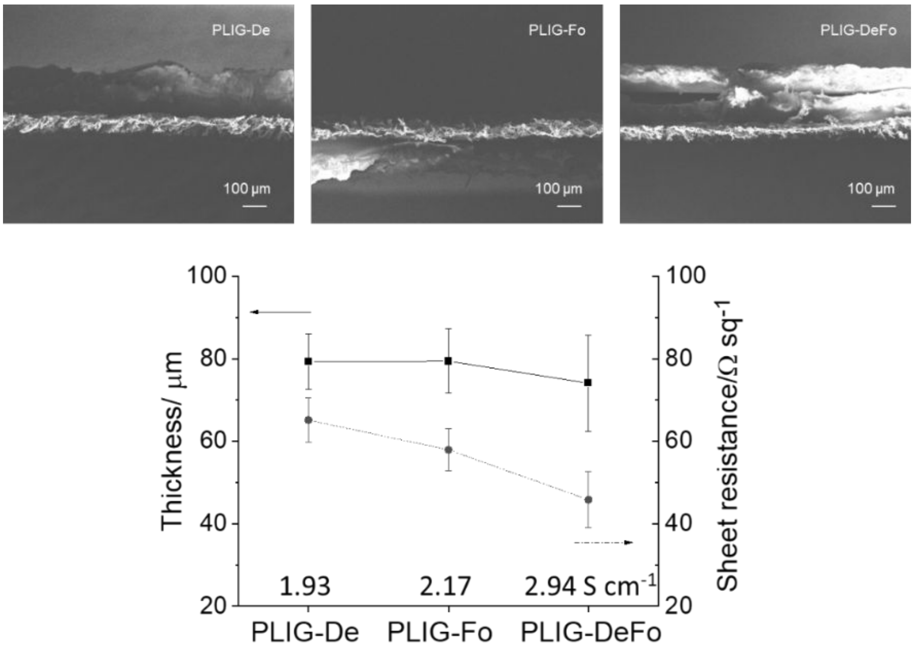

Figure S9. Cross-sectional SEM images of PLIG-De, PLIG-Fo and PLIG-DeFo layer (exfoliated from the paper by mechanical water-lifting), as well as plots of thickness, sheet resistance and calculated conductivity (σ). Thickness of each PLIG layer was estimated by cross-sectional SEM images (n=3). The electrical conductivity calculation is based on $\sigma = 1/(Rs{*}t)$, in which σ is the conductivity in S cm^{-1}, *Rs* is the sheet resistance in Ω sqr^{-1} and *t* is the thickness in cm.

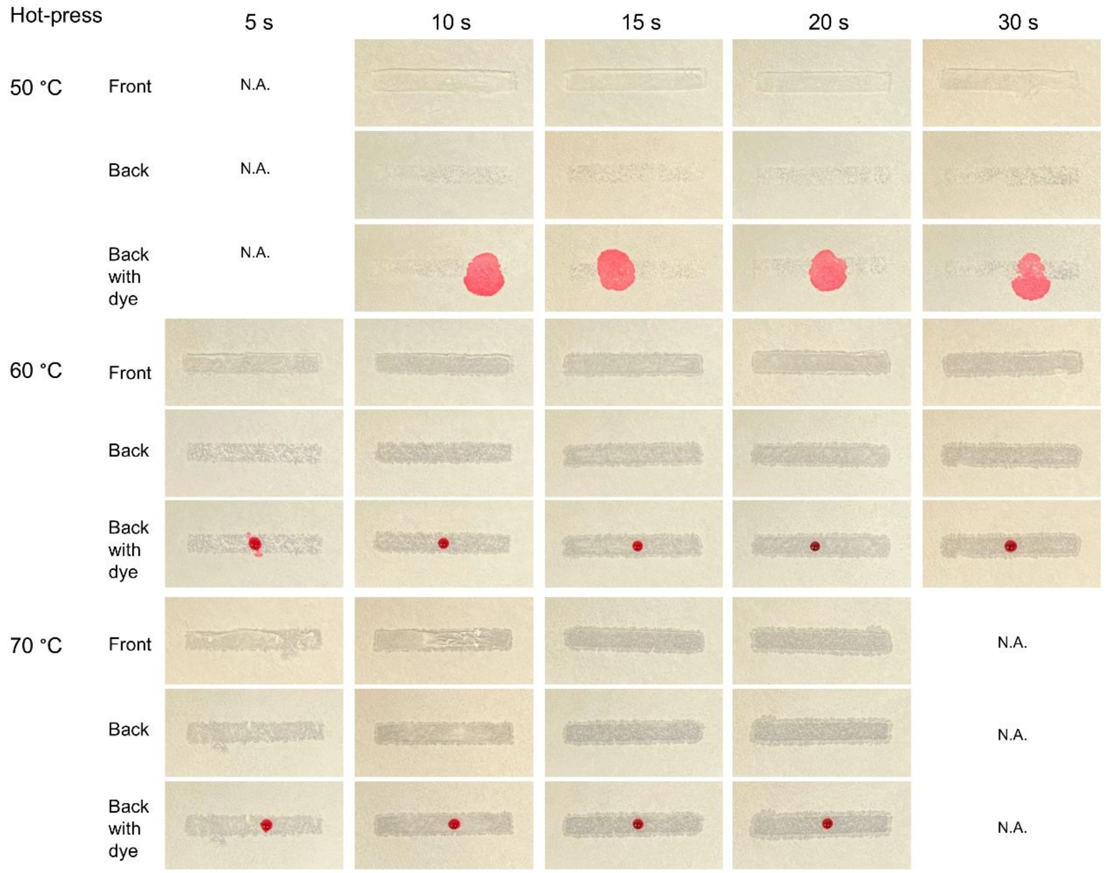

Figure S10. Optimization of hot-press patterning parameters including temperature (50–70°C) and pressing time (5–30 seconds). Digital photographs were shown in the Figure for a Parafilm-patterned paper (paper-para, through a 20×2 mm channel on the mask) in the front side (Front), back side (Back), and back side with 1 µl aqueous red dye solution dropped on the infused Parafilm area.

As shown in the Figure, hot-pressing at 50 °C cannot achieve full infusion of Parafilm on paper-back, showing a diffusive red-dye ring. At 60 °C, a clear 20×2 mm Parafilm pattern can be seen for all samples (5–30 seconds). However, a minor wick effect can be observed for paper-para fabricated at 5 seconds. When the time increased over 10 seconds, a clear round droplet can be seen on the patterned area of paper-para-back. Similarly, paper-para prepared at 70°C can guarantee the successful infusion of Parafilm through the paper with a hydrophobic region for retaining the red-dye solution. Therefore, 60°C was chosen as the optimized temperature, under which circumstance 15 seconds rather than 10 seconds was chosen to ensure the full infusion of Parafilm.

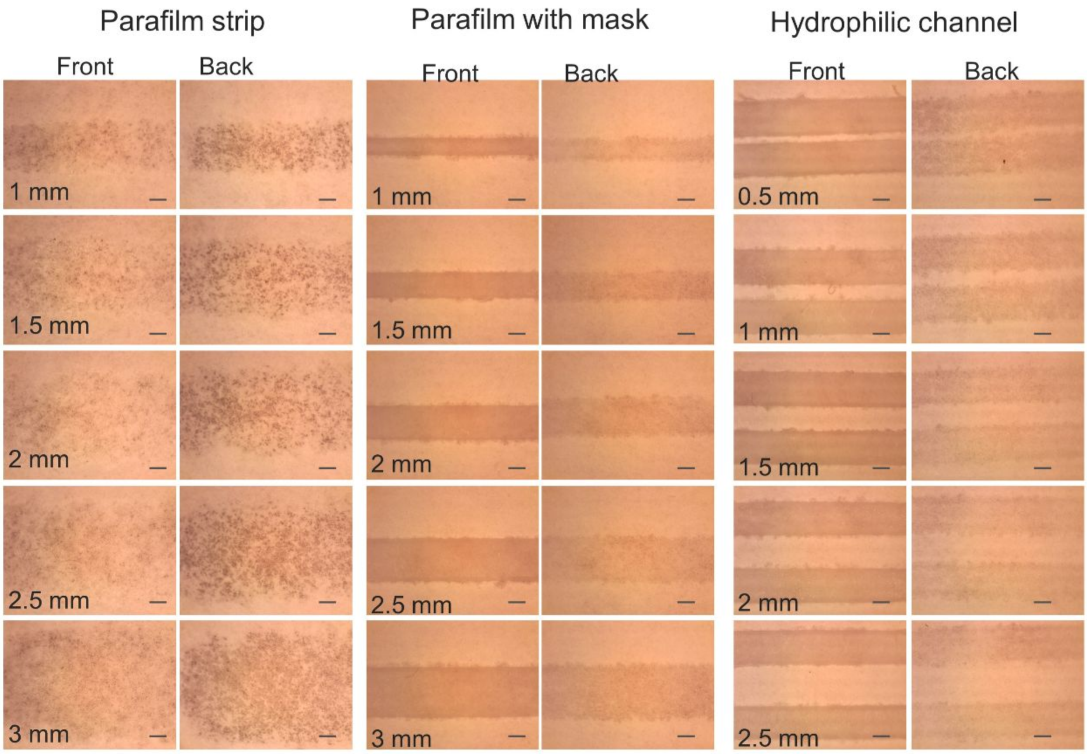

Figure S11. Optical microscopic images of various patterned microfluidic paper. The scale bar is 1 mm.

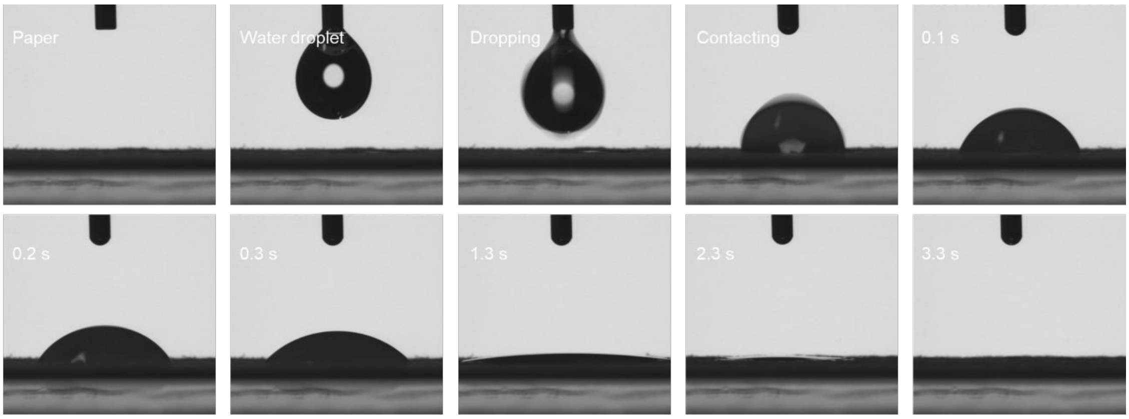

Figure S12. Consecutive record of the changes of a water droplet from needle tip to paper surface with the time interval of 0.1 second and 1 second.

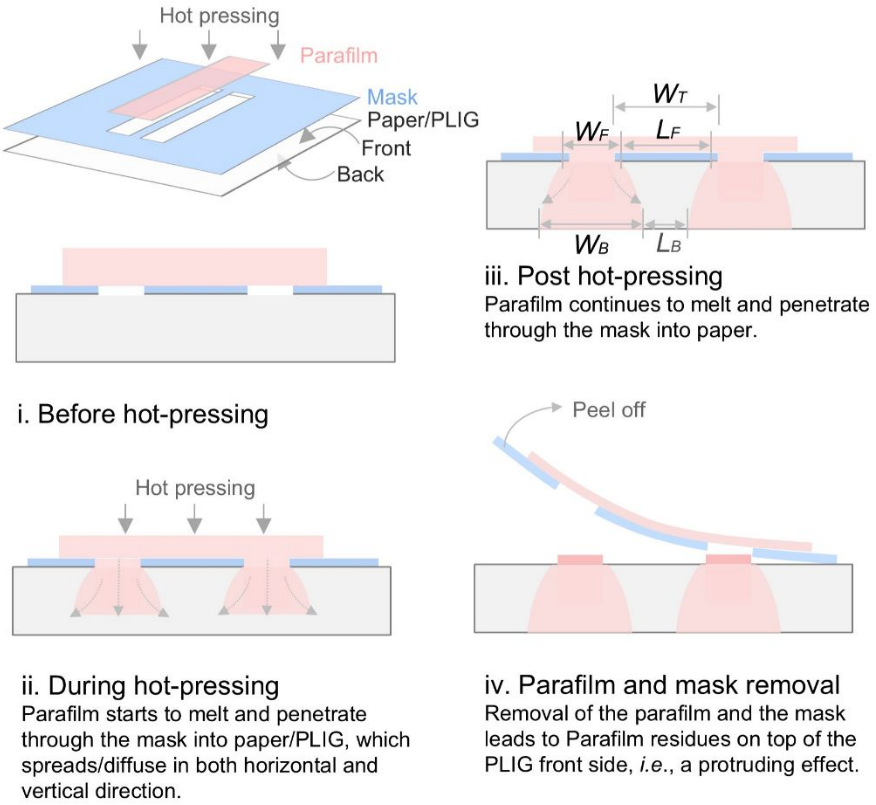

Figure S13. Schematic illustration of the diffusion and protruding effects of Parafilm on the paper-front side by hot-press patterning.

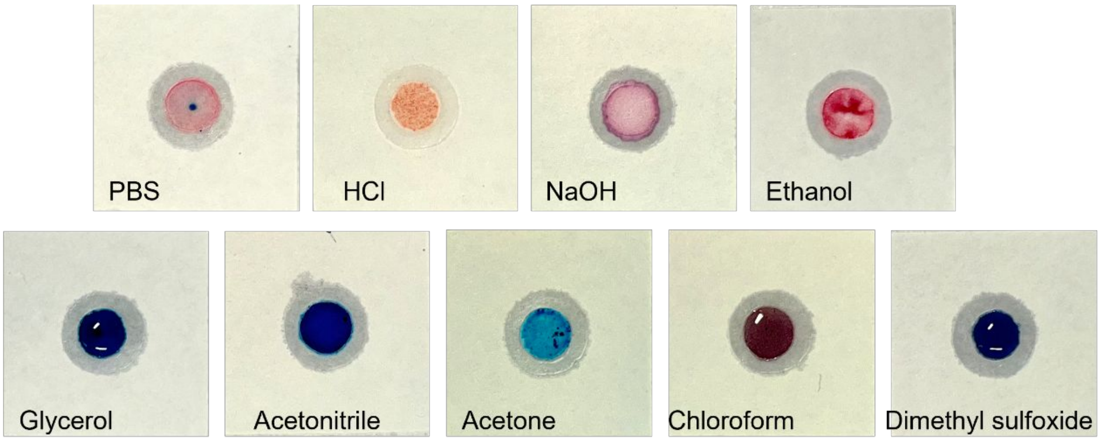

Figure S14. Solvent compatibility test with a series of aqueous solutions and organic solvents dropped in a circular hydrophilic paper region that was confined by hydrophobic paper-para, in which aqueous solutions and organic solvents are colored with various types of dyes. The digital photographs were taken after 1 hour of incubation.

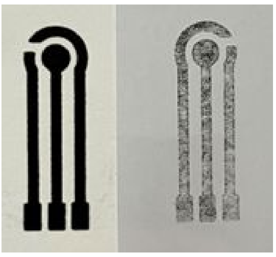

Figure S15. Digital photograph of 3-electrode PLIG after hot-pressing, left: 3 electrode PLIG, right: graphitic residues on the mask.

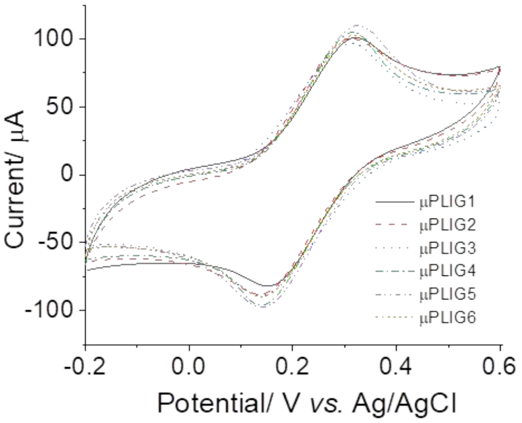

Figure S16. CV curves of six 3-electrode μPLIG in 5 mM $Fe(CN)_6^{3-/4-}$ in 0.1 M KCl against glass Ag/AgCl (3 M KCl) reference electrode.

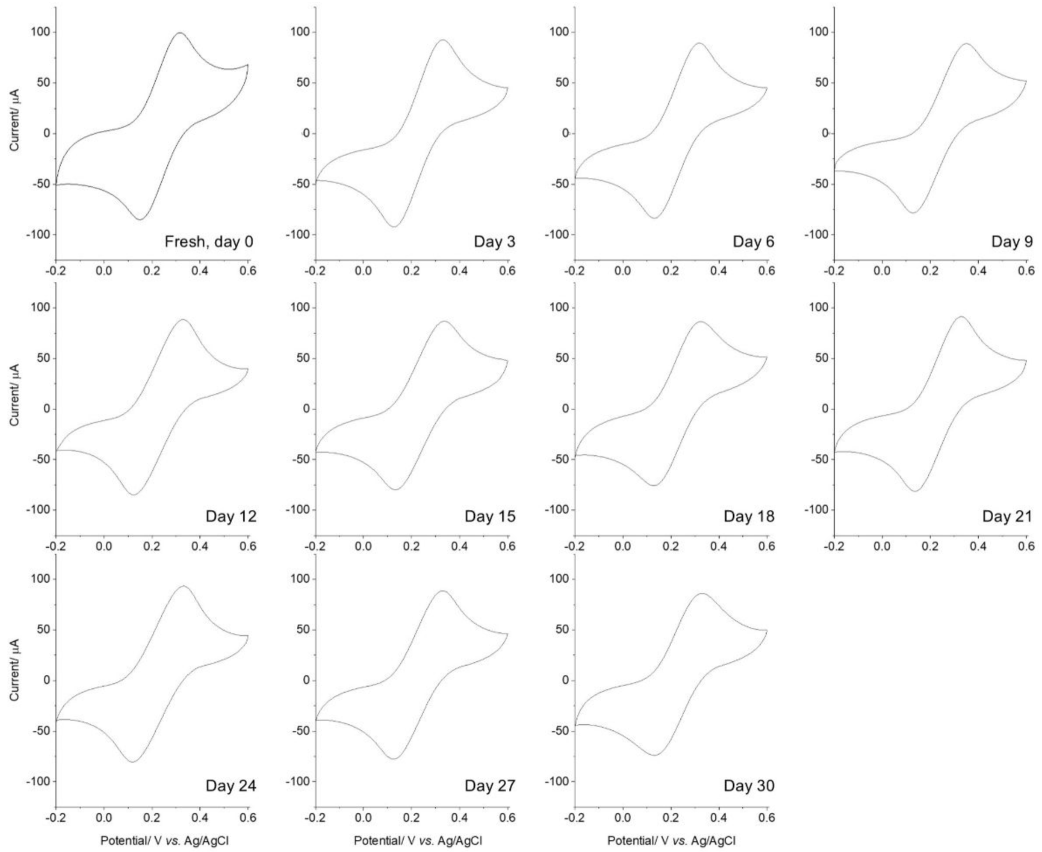

Figure S17. CV curves of the same batch of μPLIG in 5 mM $Fe(CN)_6^{3-/4-}$ in 0.1 M KCl against a glass Ag/AgCl (3 M KCl) reference electrode upon different storage times: fresh (Day 0), Day 3, 6, 9, 12, 15, 18, 21, 24, 27, and 30.

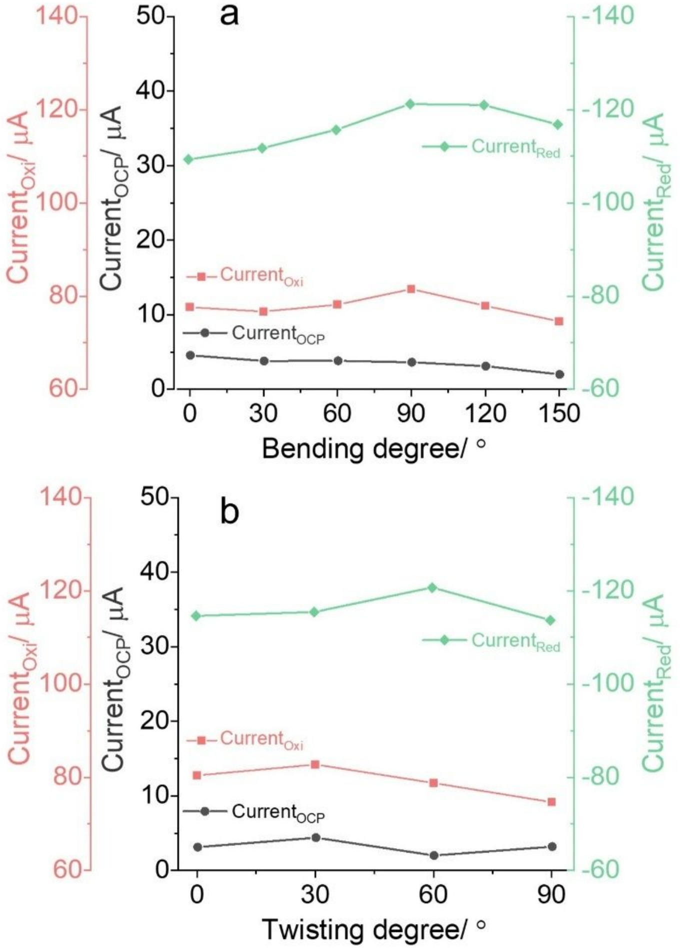

Figure S18. Plots of multi-step current values (equilibration, oxidation peak and reduction peak) upon bending (a) and twisting (b) at different angles using 3-electrode μPLIG in 5 mM $Fe(CN)_6^{3-/4-}$ in 0.1 M KCl against internal Ag/AgCl reference electrode (stencil-coated Ag/AgCl ink using a paintbrush). The multi-step CA current-time measurements were performed at open circuit potential (0.165 V) as well as oxidation peak potential (0.250 V) and reduction peak potential (0.080 V) collected from CV.

Table S3. Comparison of the µPLIG with reported microfluidic LIG.

		µPLIG	Microfluidic LIG			
**Laser processing**	Substrate	Cellulose paper (sustainable)	Polyimide (PI, synthetic polymer)			
**Microfluidic compartment**	Material	Parafilm	PDMS	PMMA/adhesive tape	Glass/acrylic sheet	PI-derived LIG/PEI
	Microfluidic fabrication technique	Hot-pressing	Photolithograph/Replica	Laser cutting platform	CO_2 laser engraver	CO_2 laser
	Integration method	Hot-pressing	Alignment/sealing	Bonding/Gluing	Adhesion bonding	Constraining
	Need for external assembly	No, intrinsic wicking for flow	Home-made chip-holder, pump	Syringe pumps	Peristaltic pump	3D-printed housing
**Microfluidic sensing devices**	Integrated into single sheet	Yes	No	No	No	No
	Flexibility	Yes	Not demonstrated	Not demonstrated	No	Not demonstrated
	Sensing demonstrated	pH sensing, lactate biosensing, Vitamin D3 detection	Multiplexed quantification of microRNAs	Alkaline phosphatase biosensing	Sucrose, NaCl, GMP, L-Tryptophan	Neonicotinoid imidacloprid and nitrate ions sensing
**References**		This work	[4]	[5]	[6]	[7]

Table S4 Comparison of the electroanalytical performance of the μPLIG platform for pH sensing (top), lactate biosensing (middle) and Vitamin D3 detection (bottom) with literature data.

Electrode	Sensitivity/ mV/pH	pH range	Reference
Electropolymerized PANi-gum /graphene oxide/ITO	-35.11	2.0–7.0	[8]
Electropolymerized PANi/carbon fiber electrode	-58.0	4.35–8	[9]
Electropolymerized PANi/PVDF fabric	-38.4	4–8	[10]
	-42.35	6–8	[10]
Pencil-drawn working electrode on paper	16.5–26.9	5–9	[11]
**Drop-casting PANi on μPLIG**	**-40.3**	**6–9**	**This work**

Electrode	Sensitivity/ μA mM^{-1}	Lactate range/ mM	Reference
LOx/PB/P-rLIG	1.21	Up to 16	[12]
LOx/PB/screen-printed graphite	0.553	0.1–1.0	[13]
CNT/TTF/LOx/Chit/screen-printed carbon fiber	0.644	Up to 20	[14]
LOx/graphene screen-printed paper	-	0.28–4.44	[15]
LOx/$FcMe_2$-LPEI/buckypaper electrode	45±6 μA cm^{-2} mM^{-1}	Up to 5	[16]
**Drop-casting PB-LOx-Nafion on μPLIG**	**0.92 (13.0μA cm^{-2} mM^{-1})**	**Up to 7.5**	**This work**

Electrode	LOD/ μM	Vitamin D3 range/ μM	Reference
GCE	0.8	2.4–350	[17]
MWCNT/GCE	17	50–1000	[18]
Cu-Ni@reduced fullerence-C_{60}/GCE	0.0025	1.25–475	[19]
GCE	0.118	5–50	[20]
SPCE	19.4	59.4–1651	[21]
**Bare μPLIG**	**1.32**	**5–65**	**This work**

Note: ITO - Indium tin oxide, PVDF - Polyvinylidene fluoride, P-rLIG - patterned reduced laser-induced graphene, PB - Prussian blue, LOx - lactate oxidase, TTF - tetrathiafulvalene, $FcMe_2$ - LPEI-dimethylferrocene-modified linear poly(ethylenimine), GCE - glassy carbon electrode, MWCNT - Multi-walled carbon nanotubes.

References

(1) Ang, S.; Narayanan, J. R.; Kargupta, W.; Haritos, V.; Batchelor, W. Cellulose nanofiber diameter distributions from microscopy image analysis: effect of measurement statistics and operator. *Cellulose* **2020**, *27*, 4189-4208.

(2) Johansson, L. S.; Campbell, J. Reproducible XPS on biopolymers: cellulose studies. *Surface and Interface Analysis: An International Journal devoted to the development and application of techniques for the analysis of surfaces, interfaces and thin films* **2004**, *36* (8), 1018-1022.

(3) Greczynski, G.; Hultman, L. Binding energy referencing in X-ray photoelectron spectroscopy. *Nature Reviews Materials* **2024**, 1-17.

(4) Liu, X.; Wang, Y.; Du, Y.; Zhang, J.; Wang, Y.; Xue, Y.; Zhao, J.; Ge, L.; Yang, L.; Li, F. Laser-induced graphene (LIG)-based electrochemical microfluidic chip for simultaneous analysis of multiplex microRNAs. *Chem. Eng. J.* **2024**, *486*, 150233.

(5) Griesche, C.; Hoecherl, K.; Baeumner, A. J. Substrate-independent laser-induced graphene electrodes for microfluidic electroanalytical systems. *ACS Applied Nano Materials* **2021**, *4* (3), 3114-3121.

(6) Wagh, M. D.; Sahoo, S. K.; Goel, S. Laser-induced graphene ablated polymeric microfluidic device with interdigital electrodes for taste sensing application. *Sensors and Actuators A: Physical* **2022**, *333*, 113301.

(7) Johnson, Z. T.; Ellis, G.; Pola, C. C.; Banwart, C.; McCormick, A.; Miliao, G. L.; Duong, D.; Opare-Addo, J.; Sista, H.; Smith, E. A. Enhanced Laser-Induced Graphene Microfluidic Integrated Sensors (LIGMIS) for On-Site Biomedical and Environmental Monitoring. *Small* **2025**, 2500262.

(8) Oliveira, R. D.; Pscheidt, J.; Santos, C. S.; Ferreira, R. T.; Marciniuk, G.; Garcia, J. R.; Vidotti, M.; Marchesi, L. F.; Pessoa, C. A. Electrochemical performance of pH sensor based on LbL films of polyaniline-gum Arabic nanocomposite and graphene oxide. *J. Electrochem. Soc.* **2020**, *167* (4), 047505.

(9) Guinovart, T.; Valdés-Ramírez, G.; Windmiller, J. R.; Andrade, F. J.; Wang, J. Bandage-based wearable potentiometric sensor for monitoring wound pH. *Electroanalysis* **2014**, *26* (6), 1345-1353.

(10) Zhao, H.; Dai, Z.; He, T.; Zhu, S.; Yan, X.; Yang, J. Fabrication of PANI-modified PVDF nanofibrous yarn for pH sensor. *e-Polymers* **2021**, *22* (1), 69-74.

(11) Kawahara, R.; Sahatiya, P.; Badhulika, S.; Uno, S. based potentiometric pH sensor using carbon electrode drawn by pencil. *Japanese Journal of Applied Physics* **2018**, *57* (4S), 04FM08.

(12) Meng, L.; Chirtes, S.; Liu, X.; Eriksson, M.; Mak, W. C. A green route for lignin-derived graphene electrodes: A disposable platform for electrochemical biosensors. *Biosens. Bioelectron.* **2022**, *218*, 114742.

(13) Kim, J.; Valdés-Ramírez, G.; Bandodkar, A. J.; Jia, W.; Martinez, A. G.; Ramírez, J.; Mercier, P.; Wang, J. Non-invasive mouthguard biosensor for continuous salivary monitoring of metabolites. *Analyst* **2014**, *139* (7), 1632-1636.

(14) Jia, W.; Bandodkar, A. J.; Valdés-Ramírez, G.; Windmiller, J. R.; Yang, Z.; Ramírez, J.; Chan, G.; Wang, J. Electrochemical tattoo biosensors for real-time noninvasive lactate monitoring in human perspiration. *Anal. Chem.* **2013**, *85* (14), 6553-6560.
(15) Moreira, J. K.; Moraes, D. D. S.; Souza, M. G.; Jantzch, G. V.; Serafini, B. F.; Fernandes, I. J.; Carreira, W. H.; Lora, P. S. Development of a low-cost graphene screen-printed paper-based electrochemical sensor with application of lactate detection: a biological biomarker. In *2023 IEEE BioSensors Conference (BioSensors)*, 2023; IEEE: pp 1-4.
(16) Hickey, D. P.; Reid, R. C.; Milton, R. D.; Minteer, S. D. A self-powered amperometric lactate biosensor based on lactate oxidase immobilized in dimethylferrocene-modified LPEI. *Biosens. Bioelectron.* **2016**, *77*, 26-31.
(17) Nallbani, A.; Holubová, J.; Sýs, M.; Tahir, A.; Vytřas, K. Voltammetric determination of cholecalciferol at glassy carbon electrode performed in water-ethanol mixture. *Potravinarstvo, volume 12, issue: 1* **2018**.
(18) Kahya, Ş. E.; Cittan, M.; Çelik, A. Electrochemical behavior of cholecalciferol on a multiwalled carbon nanotube modified glassy carbon electrode. *Cumhuriyet Science Journal* **2018**, *39* (4), 1081-1088.
(19) Anusha, T.; Bhavani, K. S.; Kumar, J. S.; Brahman, P. K. Designing and fabrication of electrochemical nanosensor employing fullerene-C60 and bimetallic nanoparticles composite film for the detection of vitamin D3 in blood samples. *Diamond Relat. Mater.* **2020**, *104*, 107761.
(20) Cincotto, F. H.; Canevari, T. C.; Machado, S. A. Highly sensitive electrochemical sensor for determination of vitamin D in mixtures of water‐ethanol. *Electroanalysis* **2014**, *26* (12), 2783-2788.
(21) Gonçalves, F. D.; Rodrigues, J. A.; Ramos, R. M. Electrochemical Sensing of Vitamin D3: A Comparative Use of Glassy Carbon and Unmodified Screen-Printed Carbon Electrodes. *Chemosensors* **2023**, *11* (12), 575.
